# Underexplored Dimensions of Emerging Indoor Photovoltaics

**DOI:** 10.1021/acsenergylett.5c03760

**Published:** 2026-02-16

**Authors:** G. Krishnamurthy Grandhi, Bruno Damien, Zeynab Skafi, Kezia Sasitharan, Hani Kanaan, Hasan Alkhatib, Sadok Ben Dkhil, Matthew J. Carnie, Marina Freitag, Thomas M. Brown, Paola Vivo

**Affiliations:** † Hybrid Solar Cells, Faculty of Engineering and Natural Sciences, 7840 Tampere University, Tampere, FI-33014, Finland; ‡ e-peas S.A., Boulevard Baudouin 1er, 19, 1348 Louvain-La-Neuve, Belgium; § CHOSE (Centre for Hybrid and Organic Solar Energy), Department of Electronic Engineering, 9318 University of Rome Tor Vergata, 00133 Rome, Italy; ∥ School of Natural and Environmental Science, 5994 Newcastle University, Newcastle upon Tyne NE1 7RU, United Kingdom; ⊥ Dracula Technologies, 2 Place Edmond Regnault, 26000 Valence, France; # SPECIFIC-IKC, Department of Materials Science and Engineering, Faculty of Science and Engineering, 7759 Swansea University, Swansea, SA1 8EN, United Kingdom

## Abstract

Indoor photovoltaics (IPVs) can significantly reduce reliance on disposable batteries in Internet of Things (IoT) devices. Yet, most evaluations use idealized lighting setups and single performance metrics, neglecting the influence of real indoor environments on device performance. This Perspective advances a deployment-centered approach: (i) realistic testing under mixed or hybrid lighting (daylight + artificial); (ii) intelligent integration that aligns absorber bandgap, series-connected cells, geometric fill factor, and power management integrated circuits with workloads and duty cycles; and (iii) IoT-ready stability assessed under the same realistic indoor scenes and light/dark sequences. We propose a compact field-to-lab pipeline, translate it into voltage-matching design rules, and use photon-to-compute metrics to link harvested power to on-device sensing and learning. The goal is low-maintenance, battery-free nodes that scale reliably in buildings, logistics, and wearable applications—ultimately cutting electronic waste.

## Graphical abstract

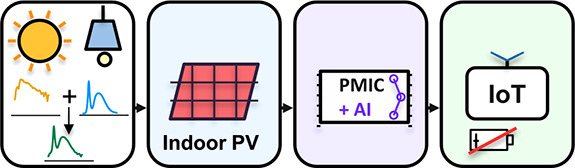

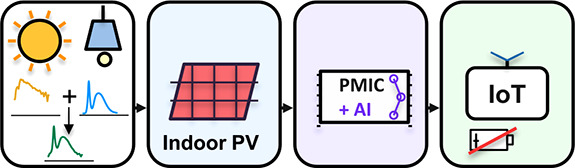

The scale of the Internet of Things (IoT) transforms indoor power into a systems-level challenge: by 2035, over 50 billion IoT nodes will operate under mixed daylight and electric lighting, where replaceable batteries drive cost, carbon emissions, and waste.^1,2^ Indoor photovoltaics (IPVs) promise maintenance-light operation by converting ambient light into useful electrical work, substantially reducing battery replacement. Yet most studies—and even recent reviews and consensus papers^3–8^ —tend to treat “indoor” as equivalent to a single artificial source, overlooking the wide variability in spectrum, geometry, and daylight fraction at the device plane in real deployments. What ultimately matters is not only cell-level power-conversion efficiency (PCE) under a reference lamp but whether a module-plus-electronics stack delivers reliable sensing, inference, and communication in the actual indoor scenes where it will be deployed.

We therefore adopt a deployment-first view: the same device-plane indoor lighting conditions that define performance should also guide integration decisions and the way we report stability. Rather than evaluating materials performance in isolation, we propose a unified, deployment-centered workflow. Operationally, we develop this thread through three critical yet underexplored dimensions. First, we root metrology at the device plane in realistic hybrid indoor lighting conditions (daylight plus artificial light) rather than idealized lamp tests, to reflect actual deployment conditions. Second, we outline intelligent integration, linking absorber bandgap, number of series-connected cells, geometric fill factor, and power-management integrated circuits (PMICs) to realistic duty cycles and photon-to-compute budgets. Third, we frame IoT-ready stability, advocating metrics and accelerated protocols expressed under the same indoor scenes and light/dark sequences used for performance evaluation. Throughout the paper, electronic-waste (e-waste) and circular design serve as a unifying theme: embedding intelligence (i.e., on-device learning) to reduce radio traffic, employing energy harvesters to reduce or replace primary (nonrechargeable) batteries, and designing materials and modules for reuse and safe end-of-life. We conclude with industry insights that translate these concepts into actionable guidance for buildings, logistics, and wearables. We proceed from defining device-plane reference indoor scenes, to translating them into PV–PMIC–storage design choices and node-level work budgets, and finally to evaluating stability under the same scene and cycling context. Figure 1 summarizes the deployment-centered workflow that links the sections below, so each section builds directly on the previous one.

**Figure 1:**
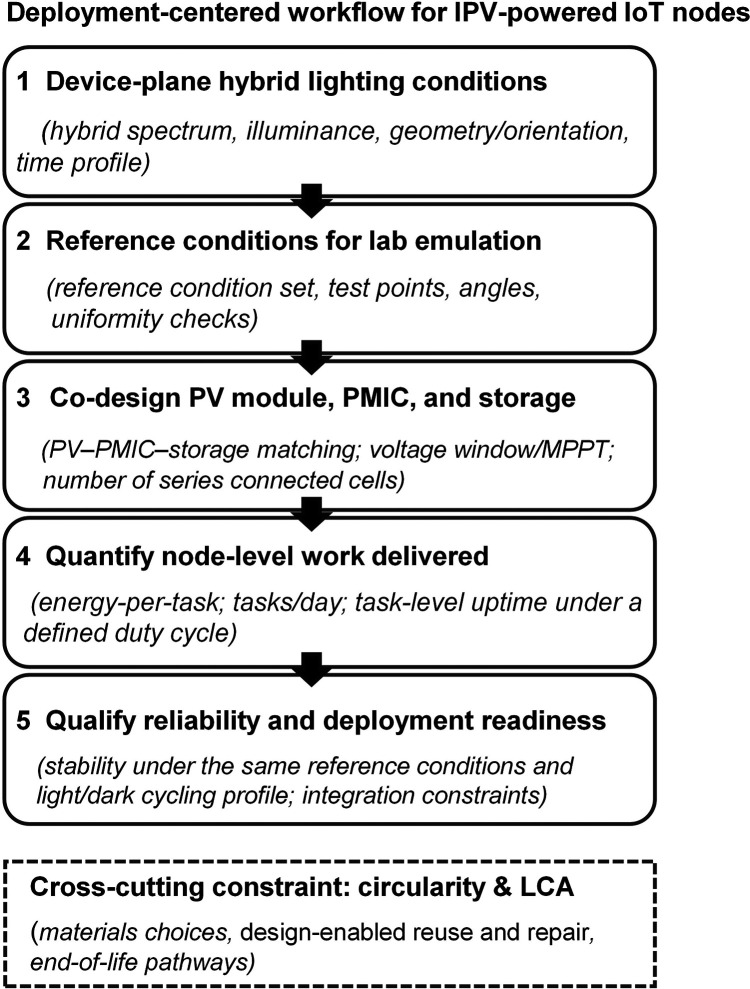
Deployment-centered workflow for IPV-powered IoT nodes, linking device-plane hybrid lighting characterization to reference lab emulation, PV–PMIC–storage codesign, node-level work metrics, and reliability assessment, with circularity/LCA as cross-cutting constraints.

## Hybrid Light Features and Characterization

The first requirement for deployment-relevant indoor PV is to quantify the device-plane indoor scene—its spectrum, geometry, and temporal variation—because this context underpins all downstream choices, from design and workloads to lifetime projections (Figure 1, step 1). Indoor-installed PV devices almost never operate under a single, static artificial source. Real spaces are illuminated by time-varying mixtures of daylight and artificial light with diverse spectra, intensities, directions, and switching patterns (occupancy, daylight harvesting, zoning). Field studies show that diffuse sunlight can supply a major share of indoor illumination during daylight hours.^9^ In smart buildings and connected environments, PV cells are often positioned in locations where both sources coexist; for instance, a sensor near a window may receive overhead LED light along with indirect sunlight. Thus, treating indoor operation as “white light-emitting diode (WLED) at varying illuminances”^10^ underestimates both complexity and opportunity.

The first requirement for deployment-relevant indoor PV is to quantify the device-plane indoor scene—its spectrum, geometry, and time variation—because everything following (design, workloads, and lifetime) depends on this context.

### Indoors, Diffuse Daylight Is Not AM1.5G Sunlight

Before reaching a device, it passes through glazing (often low-E or spectrally selective, trimming near-IR), then is reshaped by the room (multiple reflections, surface/ wall colors), and finally mixes with artificial lighting. A practical way to quantify intensity is irradiance at 1000 lx for named spectra. For example, a daylight proxy at 1000 lx carries ∼ 0.42–0.53 mW cm^–2^, whereas typical neutral-white WLEDs at 1000 lx carry ∼0.31–0.32 mW cm^–2^.^11^ Figure 2A illustrates how mixed lighting evolves at 1000 lx as daylight increases at the device plane. Figure 2B shows how spectral shape changes in a real indoor environment as scenes transition from daylight-dominant to artificial light-dominant.^9^ Scaling to realistic indoor lighting levels (50–200 lx) yields ∼26–105 μW cm^–2^ for daylight-dominant scenes and ∼16–63 μW cm^–2^ for artificial light-dominant scenes (equal lx ≠ PV-relevant irradiance).

**Figure 2:**
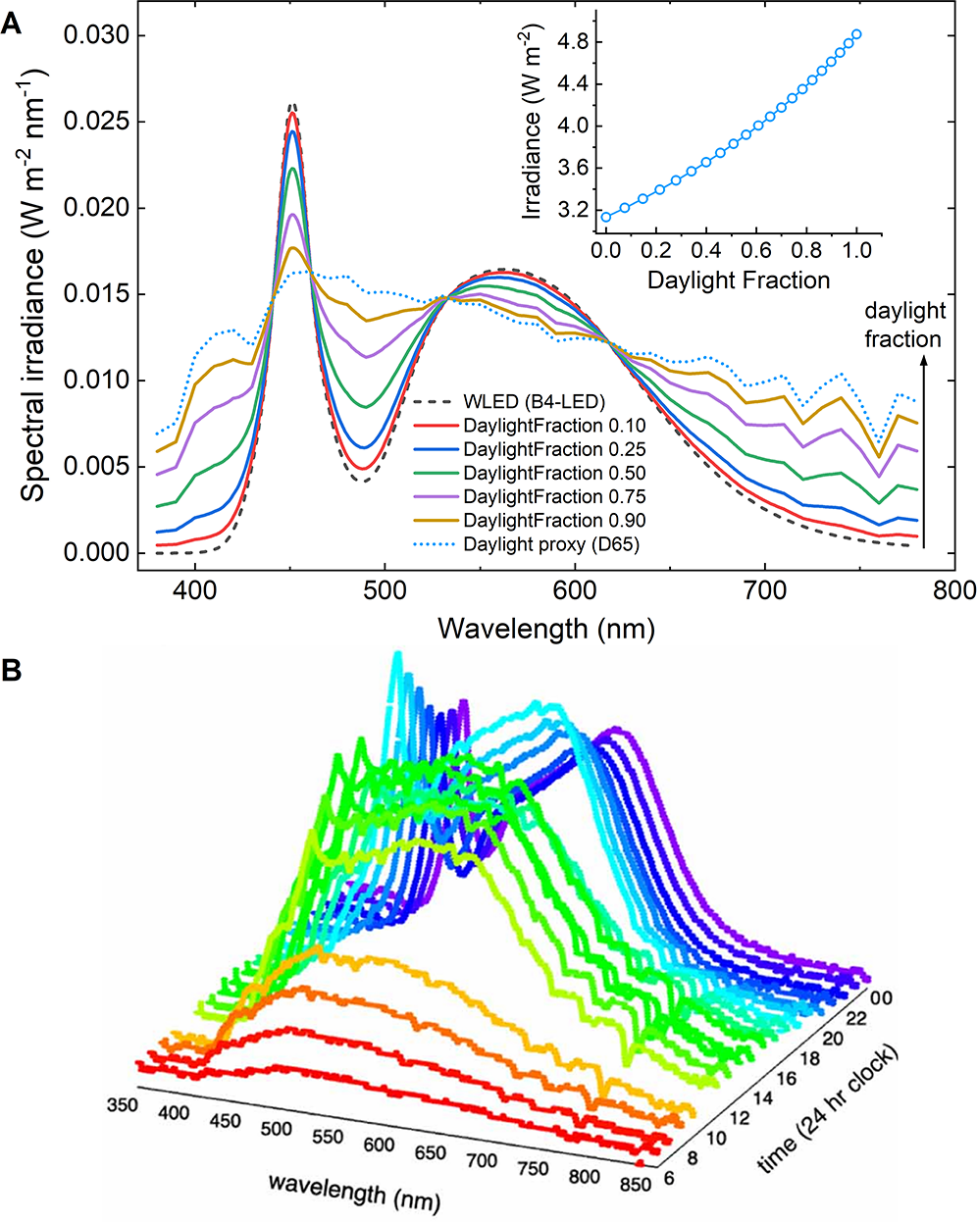
Hybrid spectra at constant illuminance and their diurnal context. (A) Spectral irradiance at the device plane for a reference WLED (CIE B4), a daylight proxy (CIE D65), and synthetic hybrid mixes (B4 + D65), each renormalized to 1000 lx. The B4–D65 mixing is used here purely to demonstrate how a measured scene can evolve as the daylight contribution increases—gaining blue and red content while retaining the LED phosphor band—illustrating that hybrid spectra are not equivalent to either source alone. Total irradiance at 1000 lx (integrated 380–780 nm) increases monotonically with daylight fraction (inset). Hybrid spectra were generated by linear mixing of a WLED benchmark SPD (CIE B4) and a daylight reference SPD (CIE D65) at varying daylight fractions, then scaling each mixed spectrum so that the computed photopic illuminance equals 1000 lx (using the CIE V­(λ) function). The corresponding radiometric irradiance was obtained by integrating the scaled spectrum. (B) Diurnal series of measured indoor spectra. Reproduced from ref 9. Available under a CC-BY 4.0 license. Copyright 2024 The Authors, published by IOP publishing. The progression shows how spectral shape shifts across the day in a real space, underscoring the need to characterize and report device-plane spectra, not lx alone, when benchmarking indoor PV under hybrid (daylight + artificial) lighting.

### Hybrid Optimum Bandgap

Indoor daylight is spectrally trimmed, weak in deep red/ NIR after glazing, and mixed with WLED blue/green peaks. Detailed-balance analyses at indoor fluxes place the single-junction optimum only slightly below the LED-only optimum (about 1.7–1.9 eV for many measured hybrid scenes^9,12^ ), rather than near ∼1.1–1.5 eV as outdoors. At low irradiance of approximately 20–100 μW cm^–2^ at the device plane (≈ 50–200 lx, spectrum-dependent), this preference for wider bandgap is amplified: short-circuit current density (*J*_SC_) falls ∼ linearly with light level, whereas open-circuit-voltage (*V*_OC_) decreases only logarithmically, and fill factor typically rises with higher *V*_OC_. As a result, performance becomes *V*_OC_- and fill-factor sensitive.^13^ This trend holds across hybrid scenes (LED-dominant, balanced, daylight-dominant), with its magnitude set by the spectral mix. A wide-bandgap reduces dark current and lifts *V*_OC_ and fill factor more than it penalizes *J*_SC_ under NIR-poor artificial-plus-daylight hybrid spectra. Consequently, wide-bandgap single-junction devices are a robust default for hybrid interiors,^9^ although the optimal bandgap shifts to 1.64 eV when harvesting diffuse sunlight exclusively.^12^

### Overview of Hybrid Light Device Testing

Controlled comparisons at a fixed illuminance of 1000 lx have shown that typical indoor sources—such as window daylight, compact fluorescent lamps (CFLs), and white LEDs (WLEDs)—deliver markedly different radiometric power at the device plane (approximately 7, 3, and 4 W m^–2^, respectively).^11^ Field measurements combining illuminance logging and periodic spectroradiometry at representative device-plane positions (e.g., walls, shelves, ceiling mounts) reveal that 50–150 lx is typical in routine indoor operation, with ∼50 lx common on vertical surfaces.^9^ Spectral content and intensity vary systematically with distance from windows, device height and tilt, and luminaire zoning or control schemes.^9,14^ Consequently, documenting the light source–device geometry at the device plane (distances to windows and luminaires, mounting height, and tilt/azimuth) alongside the measured spectrum is essential for meaningful cross-study comparisons.

In this context, material choice becomes critical. Some studies report that organic photovoltaics (OPVs) outperform amorphous silicon (a-Si),^9,14^ particularly under red or orange wall conditions. This is because OPVs maintain spectral responsiveness beyond 550 nm.^9^ Both technologies, however, perform best with highly reflective white surfaces that maximize total irradiance.^9^ Furthermore, dye-sensitized solar cells (DSSCs) have been shown to maintain performance at low light (< 13.5 mW cm^–2^) and at large angles of incidence (≥60°), outperforming Si under those conditions—behavior consistent with the low-flux, oblique illumination often encountered indoors.^15^

Validated hybrid-lighting models using ray-traced daylight and artificial light simulations (e.g., Radiance/DAYSIM), combined with measured surface reflectances, glazing transmittance, and user schedules, have been shown to reproduce real-world indoor irradiance.^16^ These models suggest that artificial lighting can contribute on the order of tens of percent of indoor irradiance during winter months, in some cases approaching ∼50%, depending on room geometry, window orientation, and node placement. Consequently, reliable IPV assessment demands consideration of combined daylight and artificial light conditions—the essence of hybrid testing. A technology-agnostic prediction framework has also been demonstrated, using spectral response and external quantum efficiency (EQE) data combined with measured indoor spectral distributions.^17^ In a comparative study of 12 commercial PV technologies (including a-Si, c-Si, mc-Si, and others), this method achieved prediction errors around ∼25%, sufficient for design trade-offs and technology ranking in realistic indoor scenes. Crucially, the study showed that device rankings shift with spectrum—technologies that perform best under WLED may be outperformed under daylight-weighted mixtures, and vice versa. This highlights the need for hybrid-lighting protocols in IPV evaluation and for identifying the most suitable PV technologies under a range of mixed-lighting conditions.

### Commercial and Research Perspectives

Commercial IPV providers have already optimized devices for ambient and mixed-light scenarios relevant to IoT. Ambient Photonics, for example, develops DSSC-based modules tailored for low-light, hybrid indoor operation, including bifacial variants that exploit reflections—a placement-aware approach aligned with hybrid reality.^18^ At the same time, the research community has started converging on best practices for artificial-light accuracy, including reference-cell calibration, spectral-mismatch correction, spatial uniformity, temporal stability, and *J*_SC_ validation against EQE integrals,^8^ which provides a strong metrological foundation for reproducible indoor PV testing. Recent standards and best-practice efforts understandably emphasize a small set of indoor reference conditions to enable cross-lab comparability.^8,19^ However, real deployments rarely experience a single “standard” spectrum or geometry: the device-plane spectrum, intensity, and angular distribution shift with daylight fraction, placement, and lighting controls. Rather than expanding standards into an unmanageably large test matrix, we propose compressing field variability into a compact set of reproducible device-plane hybrid lighting scenes that can be emulated in the lab, motivating the field-to-lab pipeline below (Figure 1, step 2).

A field-to-lab pipeline for hybrid lighting that preserves metrological accuracy.

1. **Capture field reality**: For a target use case (e.g., a wall-mounted sensor 1.5 m above floor, 2.5 m from a ceiling-mounted array of 4000 K LED luminaires, and 5 m from a window), log continuous (time-series) device-plane illuminance over representative days to weeks (e.g., 1–5 min intervals), and collect periodic spectra. Where continuous spectral logging is impractical, deploy multiple cosine-corrected lx loggers and take occasional spectral measurements to label scenes by time of day and occupancy state (lights ON vs OFF, dimming).
  Field instruments (lux loggers, spectroradiometers) are usually cosine-corrected, whereas IPV devices can show noncosine angular acceptance (e.g., due to encapsulation and mounting). With directional sources such as windows or ceiling luminaires, this mismatch can bias the PV-relevant irradiance and even the apparent spectral composition. Although a community “gold-standard” correction method has yet to emerge, a practical mitigation is to colocate a calibrated reference PV cell (or measure the DUT angular response) and explicitly report the resulting uncertainty.
2. **Define hybrid spectra and test points**: Reduce field data to a small set of spectral–temporal archetypes for the device plane: “LED-dominant,” “daylight-dominant,” and “balanced” mixtures. Test each at 50 lx, 200 lx, and 1000 lx, as recommended in IEC TS 62607–7–2:2023. These illuminance levels are not arbitrary but reflect current metrology practice for comparable IPV reporting. 50 lx represents low-light or vertical-surface operation; 200 lx serves as the baseline reference for comparability and accelerated checks; 1000 lx characterizes bright retail/supermarket exposures and can be used for optional stress testing.
3. **Emulate in the lab with traceability**: Use a two-source light engine (LED + daylight-like channel) or a programmable array to match field spectra at the device plane after accounting for room reflections and angular distributions. Use a calibrated reference PV cell together with a cosine-corrected spectroradiometer to set PV-relevant irradiance and to measure the device-plane spectrum reported.
4. **Collimated versus diffuse conditions**: For artificial-only benchmarks, quasi-collimated light improves uniformity and reduces angular mismatch. Hybrid scenes inherently include a diffuse daylight component; collimating would remove the angular structure inherent to the mixed lighting conditions. Keep one quasi-collimated baseline for cross-lab comparability and deliberately include diffuse hybrid scenes for realism (and document geometry).
5. **Report node placement and angles**: Because angular distributions differ for ceiling luminaires vs daylight, report device height, tilt, azimuth, and distances to window and luminaires. Wall color/reflectance and nearby surface finishes should be noted where practical. For wall-mounted (vertical) nodes, 0°, 45°, and 75° within the vertical plane (i.e., incidence angles relative to the device normal) can be considered as representative angles. Provide a 3 × 3 device-plane uniformity map over the active area by measuring illuminance (lx) (or PV-relevant irradiance) at nine evenly spaced points; report min/mean/max, targeting ≤ 10% nonuniformity (min/mean ≥ 0.9). For small, lab-scale device active areas (<1 cm^2^), use a 5-point (center + four) or 2 × 2 map with the smallest available sensor head and note any aperture-size limitations.
6. **Keep the artificial-light accuracy discipline**: Hybrid testing should retain the same accuracy discipline as artificial-light protocols, including traceable calibration (using a reference cell or calibrated detector), spectral-mismatch correction, spatial uniformity mapping, temporal stability checks (including flicker or PWM where relevant), validation of *J*_SC_ against EQE integrals, and reporting of the associated uncertainties.

As a concrete illustration, consider a wall-mounted IoT node in a modern open-plan office with mixed daylight and zoned LED lighting. At the device plane, the hybrid spectrum evolves both in time and space: in a south-facing windowed office it is daylight-weighted near the window and earlier in the day, but becomes LED-weighted deeper in the room and later in the day; even at a fixed time (e.g., 10:30), the spectral mix shifts systematically with distance from the window. Geometrically, wall placement introduces strong angular effects: illuminance can vary by ∼30% with mounting height, and the incidence angle that maximizes received light depends on the luminaire-to-wall distance (e.g., ∼70° at 0.6 m versus ∼34–54° at 1.2 m), motivating representative angular test points (0°, 45°, 75° relative to the device normal).^9^ Applying the field-to-lab above, one would compress the field data into three archetypal device-plane scenes (LED-dominant, balanced, daylight-dominant), then emulate them in the lab at 50 lx and 200 lx and at the same representative incidence angles. Additionally, Table 1 provides a starter library of representative device-plane indoor scenes (spectrum, illuminance, and geometry) that the community can adopt now, expand over time, and ultimately use as a basis for comparable scene-matched reporting and energy-rating metrics.

**Table 1:** Starter Set of Reference Device-Plane Indoor Scenes (Spectrum, Illuminance, Geometry, and Irradiance) Drawn from Reported Measurements/Simulations^a^

deployment context (device plane)	spectrum archetype	illuminance	geometry notes	irradiance (∼380–780 nm; reported/scaled)
Office wall node (deep/interior; ref 9)	LED dominant (cool/neutral WLED)	∼50 lx common on walls; max reported ∼150 lx in office zones	Vertical wall placement; illuminance depends on height and angle; maxima can shift with luminaire-to-wall distance (e.g., 0.6 m vs 1.2 m)	≈0.20–0.60 W m^–2^
Windowed office (midroom; ref 9)	Balanced hybrid (daylight + LED)	50–150 lx typical in routine operation (position-dependent)	Hybrid evolution depends on distance from window and time-of-day; daylight vs LED weighting shifts even at fixed time	0.20–1.05 W m^–2^
Near-window/daylight-weighted location (ref 11)	Daylight through glazing	Reference test point: 1000 lx	Geometry: nearer/facing the window (higher daylight contribution)	≈7 W m^–2^
LED-only room (controlled geometry; ref 14)	LED only (direct + diffuse components)	Wall plane can range ∼60–180 lx depending on position (brightest to dark corners)	Example room: single 4000-lm ceiling LED, no window; ceiling 2.5 m; wall illuminance varies strongly with position and luminaire distance	0.24–0.72 W m^–2^

a Irradiance values are provided as visible-band (380–780 nm) radiometric context and are spectrum-dependent. Where direct radiometric measurements are not reported in the source study, values are shown as bounded ranges (LED-like to daylight-like) using representative irradiance at 1000 lx for daylight and WLED spectra (ref 11) scaled to the listed illuminances.

With indoor scenes and spectra defined, the next question is translation (Figure 1): how do these measured conditions map to absorber bandgap, series count, geometric fill factor, and PMIC choice so that the module-plus-electronics stack (PV module, PMIC, storage, and compute/radio) delivers useful work, application-level tasks such as a sensor read, a local inference, and a 20-byte uplink—under realistic duty cycles?

## Practical Integration Challenges, Trade-offs in System Design, and Deployment Contexts for Indoor PV

Translating device-plane hybrid scene archetypes into a deployment-ready power supply requires codesign of the PV module and the power-management electronics (Figure 1, step 2). Cell architecture, module layout, and power-management strategies must be aligned from the outset to ensure best performance of the whole power supply architecture, which affects system performance, reliability, total cost of ownership, and overall carbon footprint reduction.

### Lowering Number of PV Bands (Benefits of Higher Active Area/Mechanical Area)

When considering the selection of a PV cell, the first idea is to focus on power (voltage × current) generation level in given indoor lighting environments such as 50, 100, 200, 500, and 1000 lx. PV cell designers can play with voltage and current values with the PV cell architecture. The way this power is delivered to the electronics is often neglected, yet it has a significant impact on the overall system efficiency. As shown in Figure 3, the PV cell architecture is made of several unit cells of active area, separated by “dead areas”, within the PV opening (aperture) area *W* × *L* (where *W* and *L* are the illuminated width and length, respectively). Each band is connected in series to its neighbor, so that total voltage (open circuit) is increased proportionally to the number of bands. While the current is also decreased. Unfortunately varying the number of bands does not yield a constant total power figure for a given total exposed active area. This is due to the loss of area of the interband gap.

**Figure 3:**
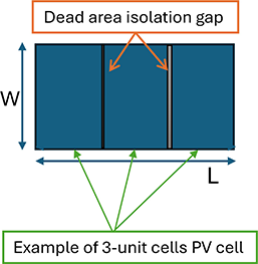
Illustration of active areas and dead areas of a PV cell. PV opening area = *W* × *L*.

As shown in Table 2, the penalty of increasing voltage is paid overall by a significant loss of active area. A good practice is to accept losses of less than 5%, which leads to preferring PV cells with 1, or 2 or 3 bands.

**Table 2:**
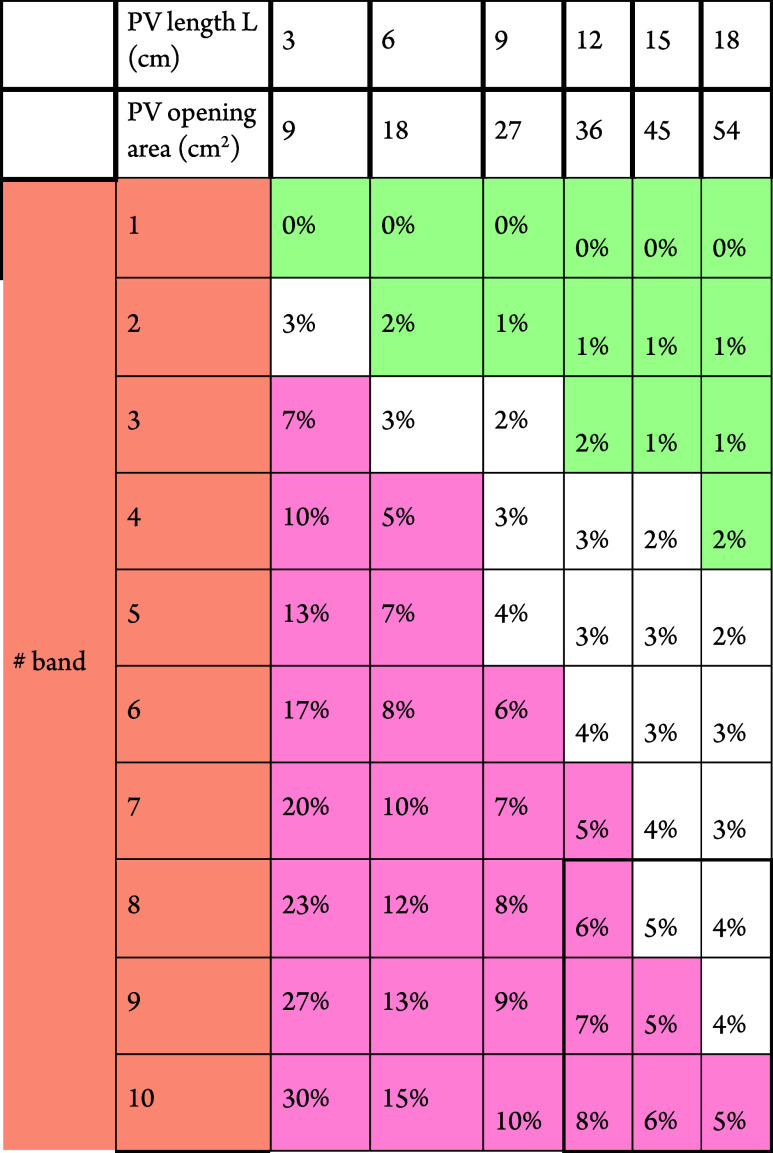
Benefit of Low Band Number in Overall Performance Going from 7/8 to 1/2/3 Bands/Increase Effective Active Area^a^

a PV opening area = *W* × *L*, with the PV width (*W*) of 3 cm and varying PV length (*L*) (the gap width (dead areas) = 0.1 cm).

In such a case, it is often necessary to convert the PV voltage by a PMIC to meet the requirements of the application and storage element requirements. For example, energy harvesting PMICs like AEM10xx or AEM00xx series from e-peas support such architectures. Note that Table 2 is a geometry-only estimate of dead-area loss from isolation gaps in series-segmented modules. Because it is purely geometric, it is spectrum-independent by construction.

### Log/Non-Log PV Behavior and MPP Strategies

As PV cells are expected to generate a certain amount of energy during use time, we will consider how much power can be extracted from it. Power = current (flowing through the PV) × voltage (elaborated at PV terminals).

It is often considered that the current generation of a PV cell is proportional to the illuminance level it is exposed to. As a first approximation, one considers it is true: photo generation is directly proportional to the number of photons landing on the PV active area (with a slope of “a”). This approximation generally holds for a fixed spectrum. Under spectral variations, such as those encountered in hybrid scenes, the same illuminance (lx) may correspond to different irradiance levels and therefore different generated currents. Secondary effects like generation saturation, electromigration resistance, and saturation of electrode efficiency may be observed at much higher illumination levels. Let us consider we are not in conditions of such extreme phenomena, so within the lighting range under consideration, the relationship is linear: *I* = *f*(lx) = *a* × lx.

The second critical parameter of the power generation equation is *V* = *g*(lx). There are two main families of PV cell technologies. One can observe that voltage at maximum power point (*V*_MPP_) may either be constant vs lx intensity (*V* = *b* × lx) or logarithmic relationship versus lx intensity (*V* = *b* × log­(lx) + *c*; Figure 4). Examples of such voltage to illuminance (lx) dependency by technologies and PV model are illustrated in Table 3.

**Figure 4:**
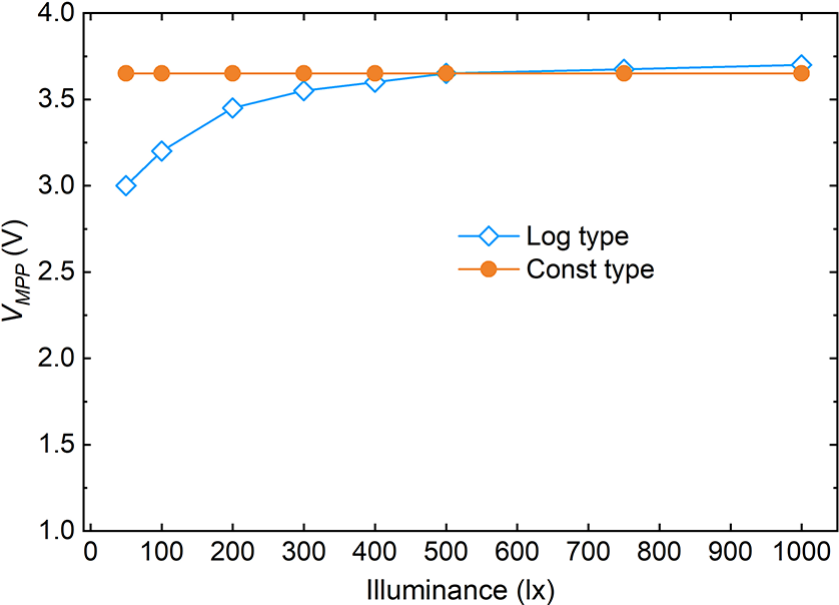
Illustration of logarithmic/constant *V*_MPP_ PV cell behavior as a function of illuminance. Here, “Const” denotes approximately constant *V*_MPP_ vs lx; “Log” denotes *V*_MPP_ that increases roughly logarithmically with lx.

**Table 3:** Various PV Models and Technologies Illustrating Performance Evolution in Time and *V*_MPP_ Dependency over Illuminance, lx (Logarithmic or Constant Type)^a^

metric	model AS	model BW	model CL	model DG
Most recent IPV technology examples				
power density (nW cm^–2^ lx^–1^@200 lx)	60	80	110	75
*V*_MPP_ type	Const	Const	Log	Const

metric	model AS	model BE	model CP	model DD
Older off-the-shelf IPV technology examples (typical)				
power density (nW cm^–2^ lx^–1^@200 lx)	40	40	35	35
*V*_MPP_ type	Log	Log	Const	Log

a Model codes are anonymized (A/B/C/D + brand initial).

### *V*_MPP_–Illuminance Behavior

Traditional monocrystalline silicon, III–V, amorphous silicon or OPV cells follow the logarithmic relationship. DSSCs and fewer organic types are often constant voltage; lead halide perovskite may be either one or the other. Real devices may fall between these limits; in such cases, the appropriate approach is to measure *V*_MPP_(lx) directly and design MPPT to track that dependence. When defining a strategy for extracting the maximum power from a PV cell, it is important to understand the behavior of *V* = *g*(lx). We often see that PV cells having a logarithmic type of *V*_MPP_ are associated with a fixed *V*_MPP_ to *V*_OC_ ratio over light intensity range. While it is not the case with constant *V*_MPP_. This has a direct effect on the *V*_MPP_ tracking strategy, and directly influences the PMIC internal behavior and product choice. AEM10xx family from e-peas are designed for tracking MPP with constant *V*_MPP_/*V*_OC_ ratio whereas AEM00xx series are tailored for maximizing power extraction at constant *V*_MPP_.

### Improvement of Performance Levels (nW cm^–2^ lx^–1^)—Case of Recent PV Model Samples

Expressing power density in nW cm^–2^ lx^–1^ as our metric normalizes IPV performance across light levels and cell sizes, providing a concise way to compare different PV architectures and technologies. In the anonymized commercial sample set shown in Table 3, IPV power density increases from ∼35–40 to > 80 nW cm^–2^ lx^–1^ over ∼3 years (under a common benchmark). The values in Table 3 are based on manufacturer measurements of anonymized commercial indoor PV products (e.g., a-Si/DSSC/OPV/perovskite) under a WLED benchmark at 200 lx (used here for comparability). They are included to illustrate (i) the range of achievable power densities under a common indoor reference and (ii) the diversity of *V*_MPP_–illuminance behavior (“Const” vs “Log”), which directly affects PMIC selection and MPPT strategy. Because both nW cm^–2^ lx^–1^ and *V*_*MPP*_ behavior can vary with spectrum and geometry, the table should not be interpreted as an interlaboratory ranking across technologies, but as an illustrative set of design-relevant behaviors under the stated benchmark. By observing this table, for the given anonymized commercial device set, we not only see the higher performance (nW cm^–2^ lx^–1^) increase but notice another interesting shift: most recent technologies often behave as constant *V*_MPP_ value whereas for older technologies the *V*_MPP_ function of lx was mostly logarithmic type. This shift is associated with the considerable efforts made in organic chemistry developments. We expect that further improvement will confirm the performance trend in the next 2–3 years, potentially bringing IPV performance closer to that of outdoor monocrystalline performance level with this metric.

It should be noted that such constant value highly differs from one technology (supplier) to another. We observe constant *V*_MPP_ values as low as 0.45 V to as high as 0.9 V, for a single band PV model. As mentioned earlier, PV manufacturers offer single, dual, triple, quadruple, or even higher number of bands. For such a diversity of parameters, the PMIC that will extract energy from the PV cell has to be flexible enough to support such a wide input range combination. Manufacturers design PMICs with this capacity.

### Benefit of Power Density Analysis for Power Supply Design

The plot of actual power generation by IPV cell normalized by lx and per square centimeter active area (Figure 5) allows us to get an idea of actual performance consistency over the illumination power. Figure 5A illustrates an IPV cell exhibiting a nearly constant *V*_MPP_ vs illuminance (lx) behavior, whereas Figure 5B shows a logarithmic *V*_MPP_–lx relationship. The preferred behavior is that the power range varies less than 10%, as shown in Figure 5A. Otherwise, simulation of system performance may become more complex. Several parameters affecting power production are embedded in the PV cell design and technology per se—such as PV aging (increase or decrease), light spectrum (warm/neutral/cold) vs PV chemistry, number of PV bands, geometry (width to length ratio), and electrode material choice.

**Figure 5:**
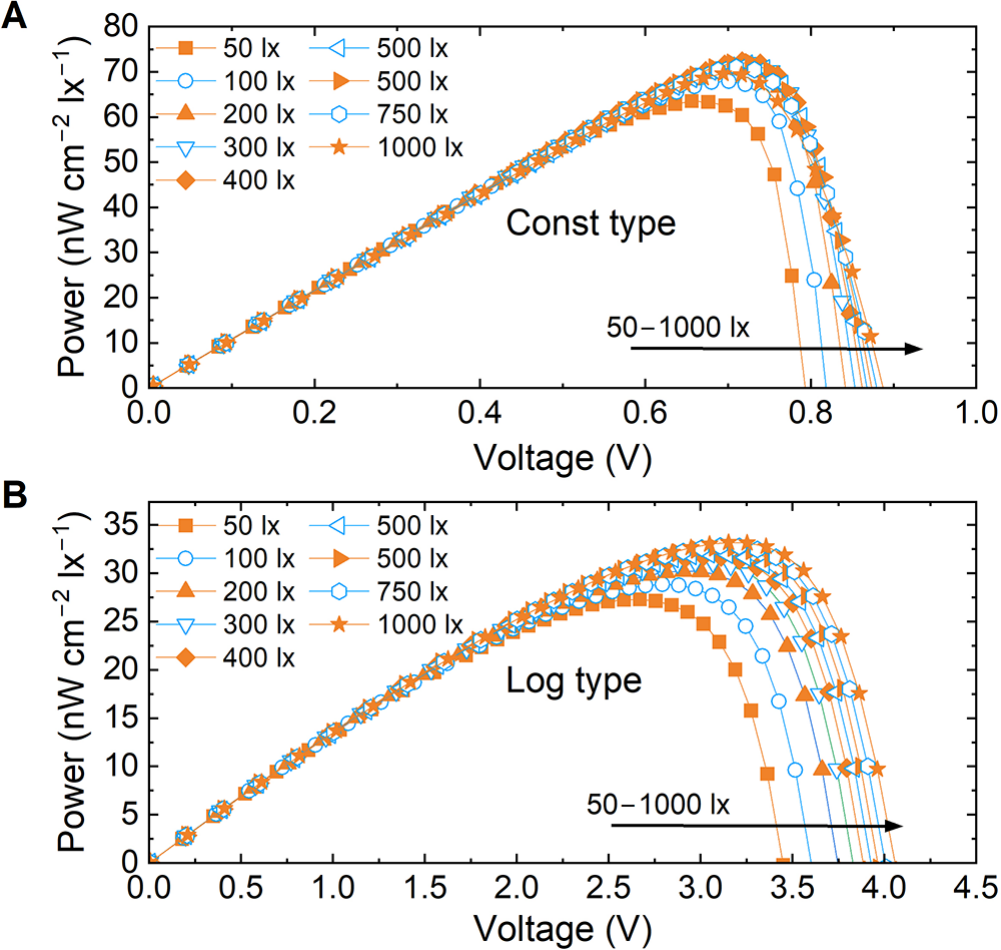
Illustration of PV cell power (nW cm^–2^ lx^–1^) production with neutral white LED source, showing (A) a cell with constant *V*_MPP_–illuminanc behavior and (B) a cell with a logarithmic *V*_MPP_–illuminance dependence.

### Role of PMICs for Compliance with Storage Elements

While PV is central to energy harvesting due to its power generation capabilities, converting this energy into usable form for electronics requires a coordinated system. The PMIC, along with the storage element, plays a key role in ensuring efficient energy transfer from PV to storage and application. There are actually two cases offered to the designer of energy harvesting solutions: case-I is when the source PV cell has voltage below the storage element (Figure 6A), case-II is the opposite (Figure 6B). Designers should consider the whole use-case and not only one situation. This includes overdischarge and overcharge of the storage element as well as low light or high light on the PV cell. In case-I (Figure 6A), the PMIC will work as a step-up converter (boost) throughout the use case conditions, in the case-II (Figure 6B), the PMIC will act as a step-down converter (buck). It is essential that these conditions are fully respected all over the operation conditions, otherwise system performance will be degraded (see Figure 6C for good practices).

**Figure 6:**
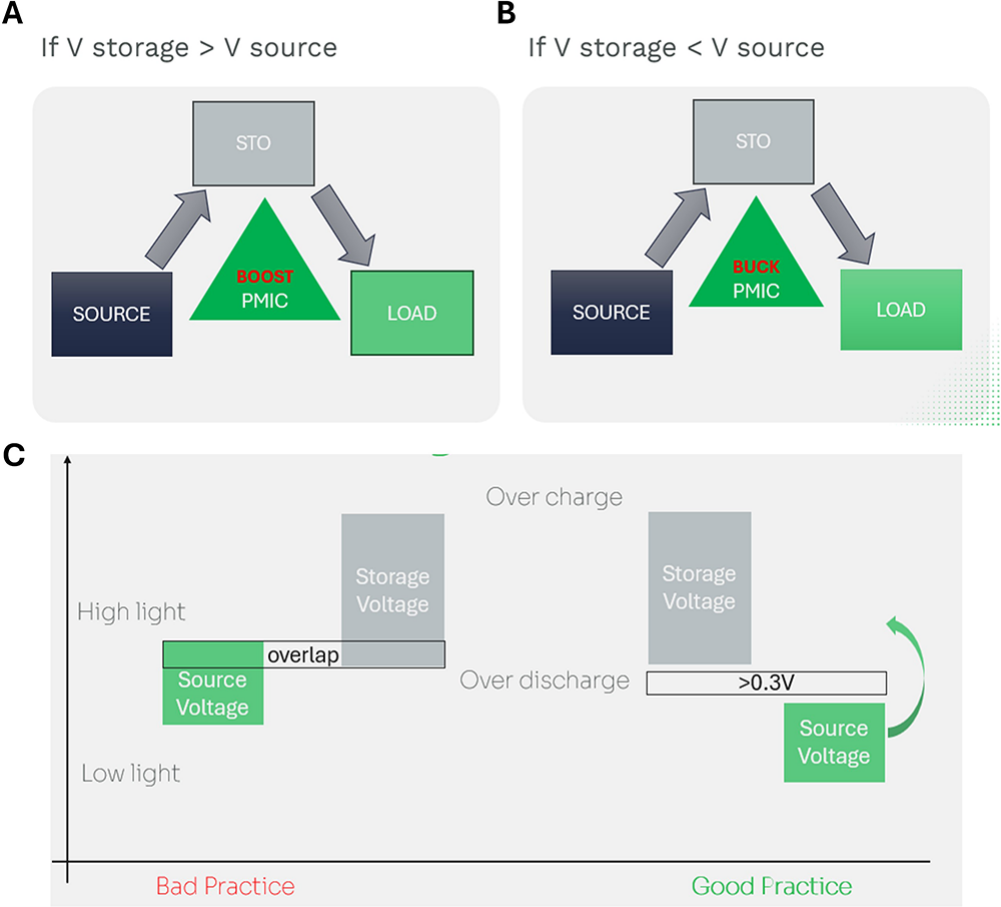
PMIC modes and voltage alignment for indoor PV nodes. (A) When V storage > V source, the PMIC operates as a boost converter; (B) when V storage < V source, it operates as a buck converter to regulate energy to the load. (C) Good practice keeps source and storage voltage windows separated across light/dark and charge/discharge cycles—avoiding overlap, overdischarge, and overcharge that degrade performance.

### Voltage Matching the PMIC—The Role of Bandgap

Because most power management ICs (PMICs) estimate the *V*_MPP_ as a fixed fraction of the *V*_OC_, series resistance and scene-dependent *V*_OC_ shifts couple directly to harvesting efficiency—reinforcing the importance of the adaptive MPP strategies discussed earlier. Put simply, the PMIC operates most efficiently within a defined input voltage range, and the module’s *V*_MPP_ must fall within this window to enable effective energy harvesting. The *V*_MPP_ of a module depends on several factors including the number of series-connected cells, the illumination spectrum and intensity, the device architecture, and ultimately, the bandgap of the absorber in the solar cell. The indoor spectral environment imposes constraints on PV module design, particularly regarding voltage matching to PMICs.

For IPV modules operating under low irradiance, matching the electrical output of the device to the requirements of the PMIC is a central integration challenge.

A detailed balance analysis of a measured mixed-spectrum indoor environment (natural + white LED) by Kay et al. indicates an optimum bandgap in the range of 1.74–1.78 eV, depending on the natural light contribution.^9^ Similar conclusions are drawn by Jarosz and Signerski, who model ideal indoor PV performance and find that bandgaps of 1.79–1.86 eV maximize power output under warm and cool white LED lighting.^12^ Their detailed balance models suggest that *V*_MPP_ values approaching 60–70% of *E*_g_/*q* (e.g., ∼ 1.1 V for *E*_g_ = 1.8 eV) are achievable under ideal low-loss conditions, however experimental studies and practical device performance often show *V*_MPP_ closer to 40–60% of the bandgap due to nonradiative recombination, contact resistance, and low-light limitations.^20^ For optimized next generation PV technologies, 0.8–1.0 V per cell is a realistic design point at 200–1000 lx.^6^

Though cold-start voltages can be as low as 180–390 mV,^21,22^ most energy-harvesting PMICs operate most efficiently when the PV input lies in the range of 0.6–2.5 V. Across this range, conversion efficiency typically increases with input voltage and load—rising from ∼55% near 0.6 V to ∼ 90% as the input approaches 2–2.5 V. Representative boost-harvester data (manufacturer datasheet; representative example) show that at ∼0.6 V the conversion efficiency is ∼70% at ∼100 μA loads and ∼85–88% at 1–10 mA.^23^ Typical midrange operation near ∼1.5 V yields ≈ 80% efficiency.^24^ These relatively low voltage requirements mean the number of series-connected cells required to meet these voltage thresholds can be reduced, which can significantly lower manufacturing complexity and cost, particularly for IPV modules.^25^ For instance, single-cell minimodules offer the lowest manufacturing cost, as they require no monolithic interconnects or laser scribing steps. However, even in such simplified architectures, attention must still be paid to series resistance (*R*_S_). While often neglected at low light levels, mixed lighting environments, where irradiance varies significantly, can exacerbate *R*_S_-related losses, especially under brighter conditions where increased photocurrent interacts with resistive elements. Elevated *R*_S_ lowers the fill factor and shifts the module’s output voltage (effective *V*_MPP_) away from the PMIC’s ideal operating point,^26,24^ degrading harvesting efficiency. Adaptive or self-tuning MPP strategies that adjust the *V*_MPP_/*V*_OC_ ratio in real time can mitigate this mismatch, particularly in dynamic hybrid lighting environments.

### Module Design—Reducing Geometric Fill-Factor

While single-cell modules can interface with PMICs at reasonable harvesting efficiencies, two or three cells in series may still be needed in certain applications to reach an optimal *V*_MPP_. However, increasing the series cell count brings its own penalties. As modules scale from individual cells to multicell layouts, one of the most significant losses arises from the reduction in geometric fill factor—the fraction of module area that is current-generating versus “dead” area taken up by interconnects and scribe lines. In energy-harvesting IoT modules, where every μW counts, even modest geometric losses can erode whole-system performance.

Geometric fill factor plays a critical role in indoor PV modules designed for IoT energy harvesting. As shown by Kay et al.,^25^ reductions in geometric fill factor directly reduce the active area available for light harvesting and hence lower the current delivered to the PMIC. These losses are particularly important in monolithically interconnected modules, where the interconnect (or “dead area”) width δ – typically the sum of P1, P2, and P3 scribe widths subtracts directly from the cell’s aperture area. Kay et al. define this relationship as

$$A_{\mathrm{cell}}=\frac{A_{\begin{array}{c} \mathrm{module} \end{array}}-\left(N-1\right)\delta L_{y}}{N}$$

where *A*_module_ is the active area per cell, *N* is the number of cells, and *L*_*y*_ is the module height. Reducing the interconnect width (δ) improves photocurrent linearly under uniform illumination, making careful module layout essential. In small-area modules, even modest geometric losses can consume a disproportionate share of the available power budget, underscoring the importance of optimizing interconnect design.

Figure 7 illustrates how increasing the number of series-connected cells in a 5 cm × 5 cm module raises both *V*_OC_ and *V*_MPP_ approximately linearly, while simultaneously reducing the current available at the maximum power point.^25^ Although the intrinsic PCE of the PV material is relatively insensitive to cell count, the combined PV–PMIC system efficiency shows a clear optimum: too few cells and *V*_MPP_ falls below the PMIC’s efficient window, too many and it drifts above it. The result is a narrow design space where geometric losses and voltage matching are balanced, typically around four to five cells depending on the absorber’s bandgap.

**Figure 7:**
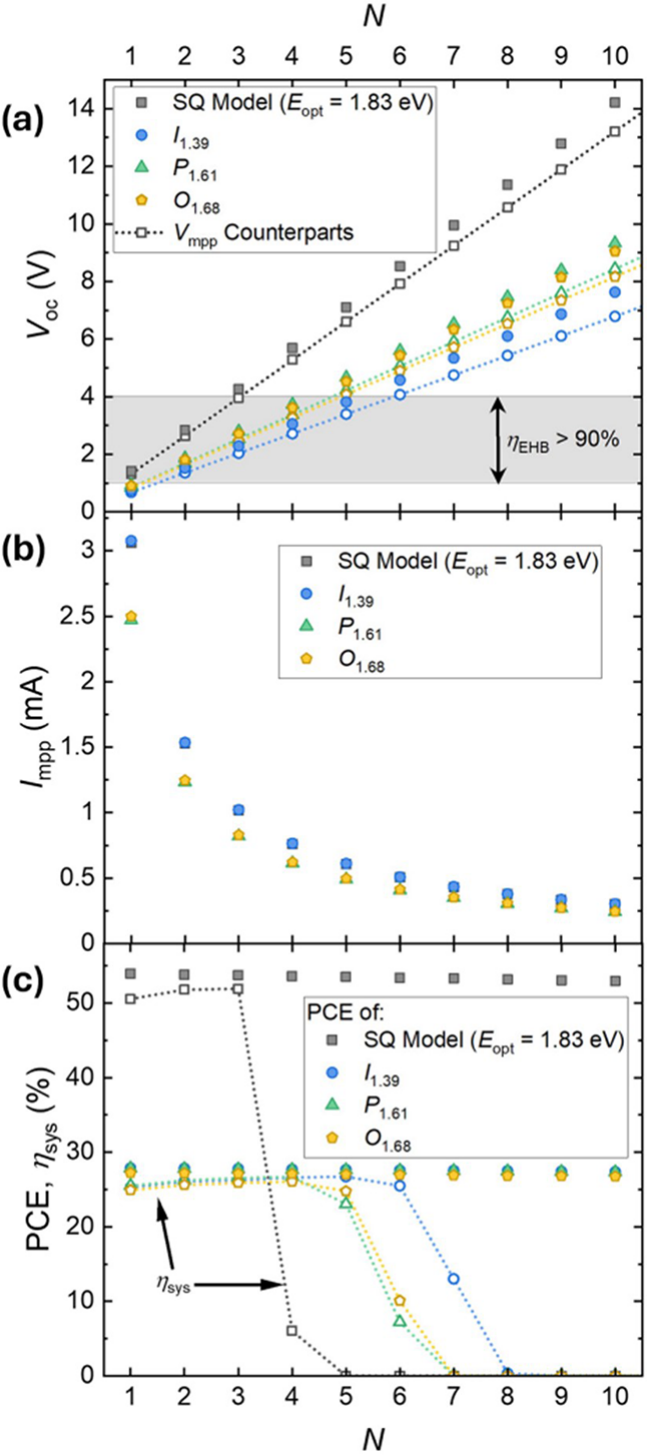
Dependence of (A) open-circuit voltage (*V*_OC_), (B) current at the maximum-power point, and (C) power-conversion efficiency of the indoor PV modules as a function of the number of connected cells (*N*) under different spectra, compared with the SQ model at *E*_opt_ = 1.83 eV. Reproduced from ref 25. Available under a CC-BY 4.0 license. Copyright 2025 The Authors, published by IOP publishing.

Several strategies have been demonstrated for maximizing geometric fill factor in thin-film PV modules, especially considering every microwatt matters in IoT energy harvesting. Rakocevic et al. used point-contact interconnections in perovskite modules to balance inactive area, series resistance, and contact resistance losses, achieving up to 99% geometric fill factor. In carbon perovskite modules (Figure 8), Meroni et al. applied a scribing method to selectively remove materials in interconnect zones, which enabled designs exceeding 90% geometric fill factor for architectures using evaporated metal contacts.^27^ Di Giacomo et al. also explored laser scribing in inverted perovskite minimodules, reporting minimodules with optimized interconnection schemes that mitigate module losses (e.g., P2 ablation strategies).^27–29^

**Figure 8:**
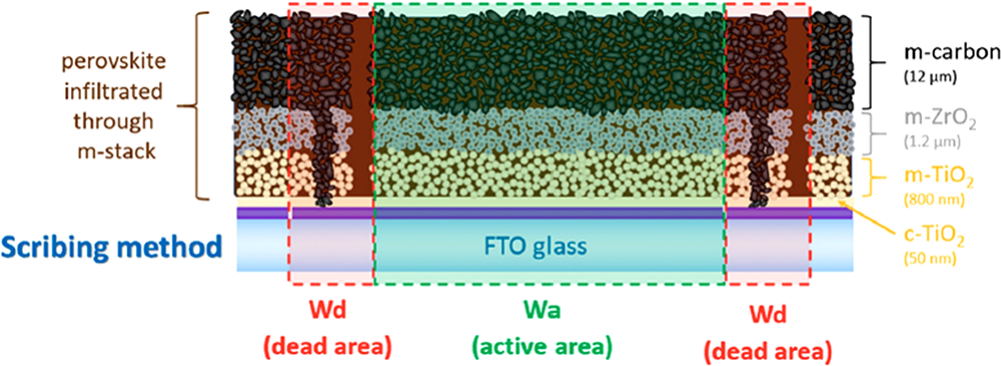
Cross-sectional schematic of a mesoscopic carbon perovskite photovoltaic cell highlighting the layered architecture (FTO/c-TiO_2_/m-TiO_2_/m-ZrO_2_/m-carbon) and the definition of the dead (*W*_d_) and active (*W*_a_) areas. Reproduced from ref 27. Available under a CC-BY 4.0 license. Copyright 2020 The Authors, published by MDPI.

Choices like the number of series-connected cells, fill factor, and matching the PMIC and storage define the voltage/current range and the conversion efficiency the node can actually use.

## On-Device Intelligence under Lux: Energy Budgets, Learning, and E-Waste in IPV Networks

The next question is how to spend that harvested-energy envelope: what sensing, local inference, and communication can be sustained under indoor ‘lux’ budgets, and how can scheduling ensure energy is spent on information rather than overhead (Figure 1, step 4)? IPVs offer an attractive alternative, yet merely replacing batteries with harvesters is insufficient. To unlock a genuinely sustainable IoT we must (i) integrate artificial intelligence (AI) for real-time energy governance and local data analytics, recognizing that at indoor fluxes radio traffic dominates the node-level energy budget (relative to sensing and computing) and that modest on-device learning can forecast irradiance and schedule work so harvested energy is spent on information rather than overhead; (ii) quantify the net energy contribution of emerging PV chemistries under ‘lux’, and (iii) design materials and system architectures that minimize e-waste across their life-cycle.

### AI-Guided Energy Integration

Indoor light is stochastic, varying on timescales from milliseconds (LED pulse-width modulation) to seconds. Naïve, static duty-cycling wastes up to 60% of the harvestable energy. Embedding machine learning (ML) at the edge enables predictive energy management (Figure 9). For instance, a long–short-term-memory (LSTM) network, quantized to 8-bit weights (6.5 kB) and executed on an ESP32 microcontroller, can forecast short-term irradiance from lx streams.^30^ Powered by a 22.4 cm^2^ DSSC array (37% PCE), this AI scheduler throttled sensing and radio tasks to maintain a 0.47 F supercapacitor between 3.5 and 5.0 V, increasing the node’s active time from 16% to 83% under a 16 h/8 h light/dark cycle (Figure 10). This principle extends across technologies; reinforcement-learning-based maximum power point tracking (MPPT) has demonstrated 6–9% greater energy capture at 200 lx for both organic (OPV) and lead halide perovskite modules compared to conventional algorithms.^31^ Crucially, on-device inference is now energetically feasible. A two-layer neural network classifying the MNIST handwritten-digit data set has been reported to require ∼ 0.95 mJ per inference on an ATmega328P 8-bit microcontroller under representative operating conditions,^32^ while a more complex CIFAR-10 (10-class natural-image) classification on a Cortex-M4F MCU consumes just 0.81 mJ.^33^ As wireless communication is the dominant energy sink in IoT (≈80% of budget),^34^ local processing drastically reduces data offloading. Transmitting a 1-byte class label instead of a 196-byte raw image saves mJ-scale per packet,^35^ more energy than the inference itself, slashing network traffic and upstream energy consumption.

**Figure 9:**
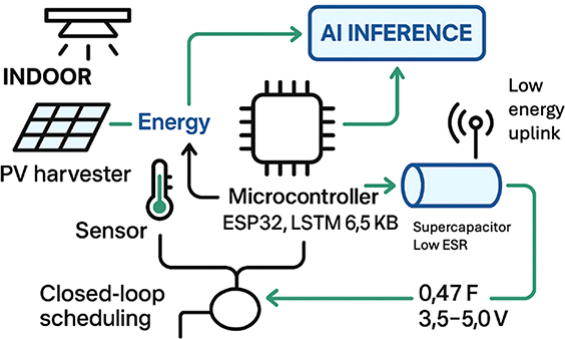
AI-guided indoor-PV node: harvested energy feeds a 0.47 F supercapacitor and an ESP32 running a compact LSTM that predicts lx and closes the loop on sensing and radio scheduling, sustaining operation around 3.5–5.0 V with minimal uplink.

**Figure 10:**
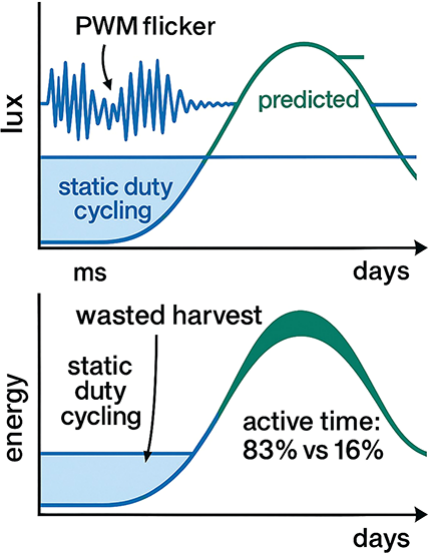
Light variability and AI scheduling.

### Energy Contribution and Photon-to-Computation Budgets

State-of-the-art DSSCs with Cu­(tmby)_2_ electrolytes deliver 141 μW cm^–2^ at 1000 lx (37.1% PCE), OPVs with PM6:Y6 nonfullerene acceptors provide 128 μW cm^–2^ (34.8% PCE), and formamidinium-cesium lead halide perovskite exceed 120 μW cm^–2^ (32% PCE).^33,36,37^ Table 4 translates these power densities into the time required to harvest the energy for a single 0.95 mJ AI inference on a 16 cm^2^ device, highlighting the viability of continuous learning under typical office lighting. These photon-to-computation budgets ultimately depend on the PV design itself (linking directly to the bandgap and series count (number of series-connected cells) discussed in the previous section): modules with higher per-cell *V*_OC_ simplify PMIC operation and enable more computational work under typical indoor lighting levels (50–200 lx).

**Table 4:** Photon-to-Compute Budgets for 16 cm^2^ Harvesters at 1000 lx^a^

PV technology	PCE@1000 lx (%)	*P*_max_ (μW cm^–2^)	time to harvest 1 mJ (s)
DSSC (XY1:L1)	38.1	141	0.58
OPV (PM6:Y6)	34.8	128	0.63
perovskite (FA/Cs)	32.0	121	0.67
a-Si:H	21.0	80	0.97

a Data compiled from refs 33, 36, and 37 .

The system-level energy contribution of IPVs must be measured not just in PCE but in computational work enabled.

At network scale, these savings are transformative. Michaels et al. showed that transmitting only inference labels instead of 196-byte raw images reduced radio energy 7-fold;^30^ while extrapolating to larger networks suggests the potential for multiterawatt-hour annual savings, actual energy reductions will depend strongly on network scale, deployment conditions, and traffic patterns. For context, even optimistic projections of such savings could approach the annual residential electricity demand of a medium-sized European country.^38^

### E-Waste and Circular Design

At the network scale, deployment-level energy decisions also have material and end-of-life consequences, because the dominant consumable in many IoT nodes remains the primary battery. The IoT boom threatens an environmental crisis, with Europe alone discarding up to 78 million batteries per day.^2^ Global e-waste hit 62 Mt in 2022, yet only 22% was formally recycled.^39^ IoT-class disposable batteries contribute significantly to e-waste with production footprints scaling to 30–110 g CO_2_-eq per cell from Li-ion battery LCAs.^40,41^ Replacing CR2032 cells with 16 cm^2^ dye-sensitized solar cell-based photocapacitor stacks (1000 lx) reduces operational CO_2_ emissions by 95% and eliminates hazardous Mn, Li and organic electrolyte leachates.^32,42^

### Material Sustainability Varies across IPV Chemistries

Modern DSSCs use glass/FTO, TiO_2_ and copper-phenanthroline mediators free of RoHS-listed elements and achieve cumulative energy demand of 6.7 MJ kWh^–1^, 4-fold below crystalline Si.^43^ Their quasi-solid “zombie” electrolytes self-heal and allow complete glass recycling; Ag grids are recoverable at >95%.^44^ OPVs boast low-temperature roll-to-roll printing but rely on indium-tin oxide and halogenated solvents; efforts toward NiO electrodes and hydrofluoro-ether inks are reducing these impacts. Lead halide perovskites deliver excellent indoor PCE but still contain Pb; tin-rich alloys and encapsulated ultrathin glass substrates cut lead mass below EU RoHS thresholds and prevent leaching but are currently unstable. End-of-life strategies must match design. FTO glass plus TiO_2_ can be regenerated after dye desorption and TiCl_4_ treatment with no performance loss, enabling closed-loop substrate reuse.^45^ Polyviologen photocapacitors with chitosan membranes degrade aerobically within 12 weeks, easing material separation.^46^ AI further promotes circularity by predicting residual capacity and signaling preventive refurbishment before catastrophic failure. Indoor energy harvesting complements these material strategies by lowering the total cost of ownership in IoT edge deployments—reducing maintenance, disposal, and raw material costs while supporting low-carbon, low-waste operation.

### Photon-Budgeted Cognition Reframes Electronics

Indoor light provides only minute, continuous energy inflows, so every computation and transmission must be planned against the small, intermittent energy harvested from photons. ‘lux’ becomes a currency governing every CPU cycle and radio burst. High-voltage (>0.9 V) indoor harvesters already support sub-mJ inference at kilohertz duty cycles. The next leap will couple federated learning—distributing model updates at ≤0.2 mJ per round, with ultralow-leakage photocapacitors (<100 mV overnight drop) and lead-secure perovskites. Standard metrics such as “energy-per-inference” and lx-specific lifetime carbon accounting should accompany IEC TS 62607–7–2 to guide cross-disciplinary optimization.

To realize a future of intelligent, autonomous, and sustainable indoor electronics, the research community must move beyond optimizing cell-level PCE. The critical frontiers are systemic and interdisciplinary:

1. **Standardized metrics**: The IEC TS 62607–7–2 standard for low-light testing is a crucial first step. We must now develop system-level metrics like “energy-per-inference” (μJ/inf) and “lx-to-decision efficiency” to benchmark AI-ready IPV systems.
2. **Integrated Power Management**: Reducing the overnight voltage drop (≈500 mV) in large-area photocapacitors is essential for robust dark operation. This requires engineering solid-state electrolytes and passivating interfacial recombination pathways.
3. **Energy-Aware AI**: Developing federated learning and neuromorphic computing architectures that operate within the submilliwatt budgets of 200 lx harvesters will be key to deploying distributed intelligence without a massive energy penalty (Figure 10).
4. **Circular-by-Design Materials**: Full LCAs for emerging OPV and lead-reduced perovskite materials are needed to guide the development of truly sustainable technologies that are nontoxic, resource-efficient, and designed for disassembly.

By codesigning materials, devices, and algorithms, we can transform the ubiquitous photons in our built environment into perpetual, actionable intelligence. This approach not only solves the power problem for the IoT but also addresses its looming e-waste crisis, paving the way for a truly smart and circular electronic ecosystem.

An energy-aware node must also be time-aware: performance and lifetime hinge on how materials and modules behave under the same light/dark sequences and partial shading seen in the field. We therefore express stability in those very realistic conditions (Figure 1, step 5).

## IoT-Ready Stability

The operational stability of emerging IPVs is a critical determinant of its adoption in self-powered IoT systems. Modules commercialized for use outdoors endure UV/IR radiation, temperature extremes, humidity fluctuations, and mechanical stresses from hail and wind, conditions that demand heavy encapsulation and robust construction to reach the 25-year performance warranty associated with commercial panels.^47^ In contrast, IPVs operate under much lower irradiance (also mostly lacking UV/IR components), as well as temperature and humidity variations. This milder environment relaxes encapsulation requirements^6^ but introduces a different challenge: stress factors vary widely between applications,^8^ creating diverse lifetime expectations and necessitating application-specific stability protocols. Industrial and infrastructure systems may demand lifetimes similar to the ones for outdoors, while consumer IoT devices often operate for only 5–10 years before replacement.^7^ Manufacturers such as RICOH state in their specification sheets that their solid-state DSSC solar cells maintain high power output over a wide operating temperature range (−30 to 60 °C), enabling their use in refrigerated and frozen environments as well as in indoor environments.^48^ As another example, Lightricity provides fully PV-powered wireless sensor platforms for monitoring the location, temperature, movements of assets, and air quality, designed to operate across different environments with temperature ranges of −40 to 85 °C or 0 to 45 °C, and with PV component area sizing that enables operation from 80 to 200 lx and above.^49,50^ Dracula Technologies designed OPVs to deliver energy starting from 50 lx.^51^ In addition, wearable IPVs impose further constraints. Devices integrated into garments or skin-contact sensors must maintain performance under flexing, stretching, washing, and continuous body interaction. For biomedical applications, the IPV device’s biocompatibility is also a requirement.

### Differential Aging

Hydrogenated amorphous silicon (a-Si:H) is currently the industry-dominant technology for IPV due to a suitable bandgap to harvest visible light and high shunt resistances as well as good stability. Its PCE can reach 30% in lab-scale devices, while the best-performing commercial devices are in the range of 7–16% under 200 to 1000 lx illumination.^6,8^ Despite the remarkable efficiency reached in the lab (30–45%), new-generation PV technologies lag behind a-Si:H technology in stability. Figure 11A shows that, after 7 months of shelf life tests, the two a-Si:H PV cells under study did not age, whereas the PCE of DSSCs measured at 200 lx dropped by 50% or more.^52^ It is notable that the drop is much more significant when the same cell is measured under 200 lx compared to when it is measured at standard test conditions (STC), i.e., 1 Sun. Similar differential aging rates have also been observed for perovskite technology.^53^ The larger performance drop at 200 lx versus STC underscores why scene-matched testing is essential for credible warranties indoors. Defects created during aging are felt on performance much more at the low photocurrents generated under indoor illumination compared to at 1 sun. Understanding their origin, the effect of environmental stressors, and how to limit their concentration through intrinsic (e.g., materials and interface) as well as extrinsic (e.g., encapsulation and permeation barriers) engineering is crucial for the technology to enter commercial markets.^6^

**Figure 11:**
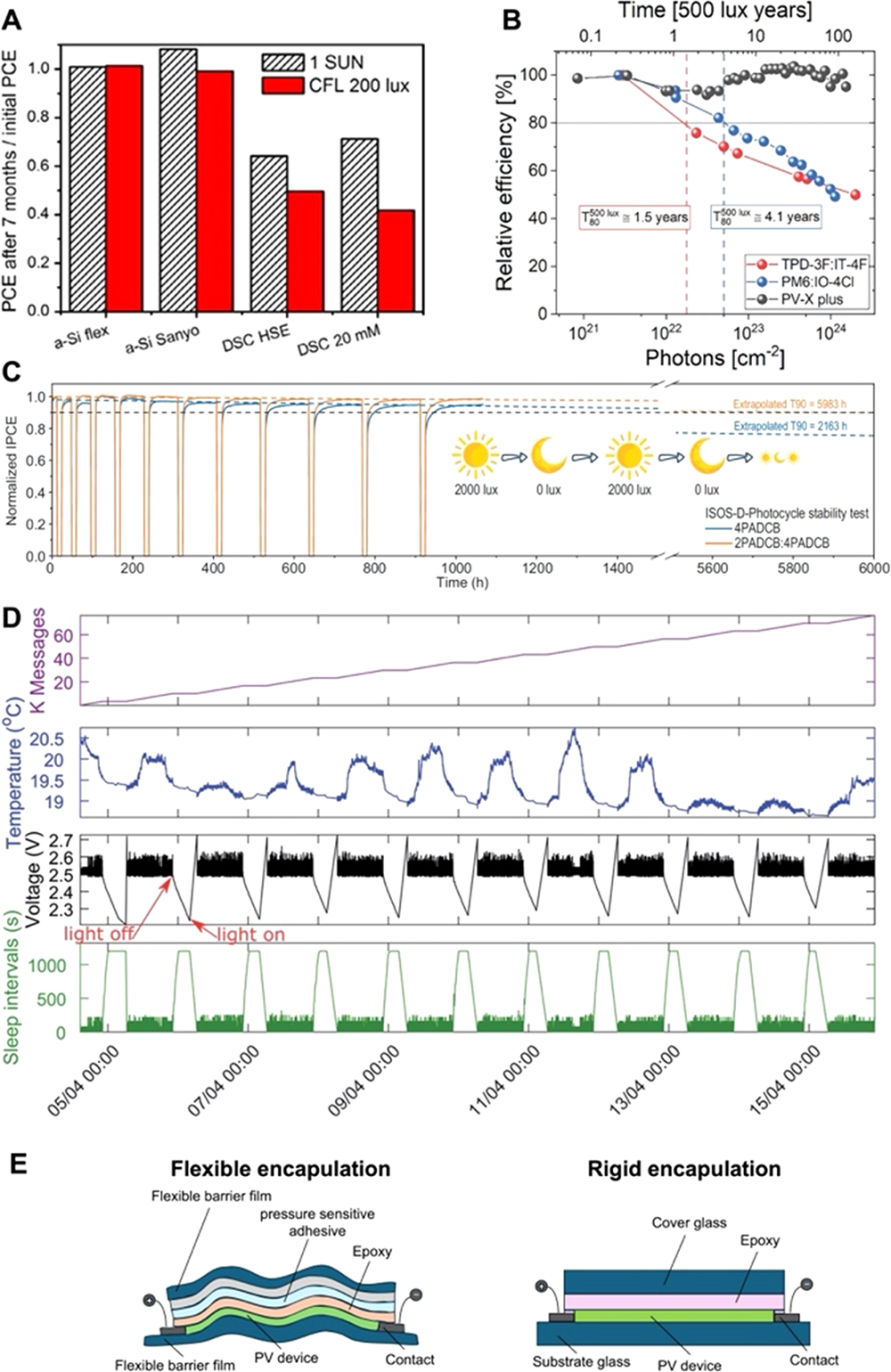
(A) Ratio of PCE after 7 months of ambient shelf life to initial PCE for a-Si:H and DSSC devices, measured under standard test conditions (dashed bars) and low indoor illumination (solid red bars). Reproduced with permission from ref 52. Copyright 2015 Elsevier Ltd. (B) Relative efficiency at 500 lx for OPV cells with different absorbers, plotted as a function of incident photon dose under 50000 lx aging test. Dashed lines mark *T*_80_ lifetimes, converted to equivalent photon doses at 500 lx. Reproduced from ref 54. Available under a CC-BY 4.0 license. Copyright 2023 The Authors, published by Wiley-VCH GmbH. (C) ISOS-D-Photocycle stability test of perovskite PV cells with two different self-assembled monolayers. The cycles consisted of 12-h periods with light-to-dark duty cycles of *n*:1, where *n* represents the dark period length before each illumination phase. The applied conditions, 12­(*n* + 1)-hour cycles (12 h light/12 h dark, 24 h light/12 h dark, 36 h light/12 h dark), simulate diurnal operation. Reproduced from ref 55. Available under a CC-BY 4.0 license. Copyright 2025 The Authors, published by Oxford University Press on behalf of China Science Publishing & Media Ltd. (D) Wireless sensor node powered by five serial DSSC PV cells, tested over 12 days under simulated light intervals of 16 h of illumination (1000 lx) followed by 8 h of darkness (10 pm–6 am, indicated by red arrows). Reproduced from ref 32. Available under a CC-BY 3.0 Unported License. Copyright 2020 The Royal Society of Chemistry. (E) Schematics of flexible encapsulation stacks for OPV and glass-glass encapsulation for perovskite PV devices.

### Accelerated Testing

To gain insight into the physical mechanisms governing IPV degradation, ISOS-style testing protocols should be established for indoor operation. Furthermore, the development of indoor-specific qualification standards, comparable to IEC 61215 used for outdoor PVs, will also be important because IPVs operate under fundamentally different environmental conditions (e.g., low and spectrally narrow irradiance, different light/dark cycling patterns depending on the function of the building, partial shading due to furniture and position of people, more limited but persistent thermal and humidity variations) and exhibit unique failure modes accordingly (as well as due to their heightened sensitivity to defects arising in time).^52,53^ Although indoor environments are generally more controlled than outdoor settings, they can vary significantly with geographical location, building usage, and deployment position (e.g., under furniture, on walls, or ceilings, near windows, in more humid cellars vs drier laboratories), which may impact device performance and stability.^8^ Accelerated stability testing remains an underdeveloped aspect of IPVs.^8^ As an example, Epishine has developed seven customized accelerated stress tests using thermal, light, mechanical and electrical stressors, and has defined a pass criterion. These include temperature–humidity exposure (−20 °C/12% RH for 3000 h with no power loss, and 55 °C/85% RH for 2000 h with ≤20% power loss), high-illuminance warm-white LED exposure (42000 lx for 3 h day^–1^ over 100 days at 22 °C and 45% RH, with ≤5% power loss), and 10000-cycle bending tests (1 cm radius), as well as 5 m drop tests, with no power loss as the pass criterion.^56,57^ Laboratory-scale investigations have advanced the development of photo- and thermally stable OPVs by employing accelerated light-exposure testing protocols, in which degradation behavior is correlated with the cumulative photon dose (see Figure 11B). Under 50000 lx LED illumination—equivalent to more than 110 years at 500 lx—devices showed only <4% efficiency loss after over 11000 h.^54^ An ISOS-D-Photocycle protocol, which alternates between light and dark phases to simulate day/night cycles, was also employed to evaluate the stability of the perovskite PV devices. Figure 11C shows that during accelerated aging tests, with light intensity varying between 2000 and 0 lx, the cells exhibited a *T*_90_ lifetime of approximately 6000 h.^55^ Additionally, another often-overlooked source of instability in IPV is partial shading, which primarily affects large-area devices. Shading can push the affected region into reverse bias, potentially causing irreversible damage to the modules. This leads to rapid degradation including microstructural changes in the material under STC.^58^ The knowledge gap regarding this type of degradation and its solution (e.g., bypass diodes) should be explored in the future under indoor illumination where photocurrents are orders of magnitude lower. Although the IPV space has started to look into indoor stability testing, it still lacks enough field tests indoors and statistical data that link accelerated aging with long-term operation in real environments.^8^ Key system-level metrics, such as continuous operation under light/dark cycling, voltage stability, mechanical durability, and overall uptime, are rarely reported. For example, Figure 11D shows that self-powered sensor’s operating voltage powered by DSSCs over 12 days in time depends on the lighting environment: most stable in factories, moderately variable in offices, and highly variable at home due to mixed lighting.^32^ The effect of integrated codesign of energy generation, storage, and power management as well as varying illumination and other environmental conditions on the stability of the PV unit remains limited, constraining the practical deployment and scalability of IPV-powered IoT systems. Providing credible stability data and performance warranties will enhance confidence in the manufacturer. To achieve this, designing reliable accelerated tests for estimating whether IPV devices can operate for the full-service lifetime of the products they are intended to power under specific operating conditions becomes important. To enable meaningful comparisons and to more precisely link stress-test results with actual operating lifetimes, the IPV field needs standardized stability protocols that are adopted across the community. In addition, field tests on IPV devices are required to establish practical relationships between degradation observed in stress tests and degradation during real-world operation in different environments, allowing confident lifetime projections. Developing these relationships will take time, as they will depend on the specific materials and technologies under development.

### Stability Enhancement Strategies

Improving device lifetimes mainly relies on two main approaches: increasing the intrinsic stability of the active materials and designing effective encapsulation to mitigate environmental degradation. Often, the strategies that minimize defect densities during fabrication are also effective in preserving these low levels throughout device operation. Passivation of films—both in the bulk, through dopants or additives in the precursor solutions, and at interfaces, via interfacial engineering or the introduction of interfacial layers—has proven highly effective and is even more critical for indoor devices than for those designed for STC. Furthermore, reducing shunting paths that enable undesirable charge recombination can be achieved by employing thicker absorber layers and improving the quality of both the films and their interfaces. A direct approach to mitigating extrinsic instabilities arising from environmental stress is through effective encapsulation. For indoor applications, the operational environment typically imposes milder stressors, and the expected device lifetimes can be shorter owing to the limited lifespan of the host products. As a result, encapsulation for indoor devices is often assumed to require less stringent protection and lower cost; however, dedicated studies are needed to validate and optimize encapsulation strategies tailored to indoor environments. In certain devices, encapsulation design must also address the containment of materials such as DSSC electrolytes or lead compounds from lead halide perovskites to prevent leakage. Whereas glass remains the substrate and permeation barrier of choice for outdoor installations, owing to its unmatched durability and barrier properties (with a WVTR below detection limits of 3 × 10^–7^ g m^–2^ day^–1^) (see Figure 11E, right), for indoor applications, the advantages of flexibility make device fabrication on flexible substrates particularly attractive, leading many companies to pursue IPV development in this format. Its lightweight and adaptable nature, along with benefits arising from roll-to-roll manufacturing enables a more seamless and less bulky integration. However, commonly used polymer films such as PET and PEN exhibit relatively high WVTR (ranging from 1.3–3.5 g m^–2^ day^–1^ for PET and 0.5–1.5 g m^–2^ day^–1^ for PEN), which are not suitable for a stable technology. Thus, permeation barriers need to be applied. The effectiveness of these barriers improves with increasing layer number and complexity, though this also raises fabrication costs. Recent studies indicate that flexible OPV devices can achieve long operational lifetimes under indoor illumination, even with moderately effective encapsulation (e.g., *T*_80_ of 74 years under 500 lx when encapsulated with a moisture barrier film with a WVTR of 3 × 10^–3^ g m^–2^ day^–1^ using a pressure-sensitive adhesive).^54^ In contrast, OPVs for the outdoors require much stricter moisture and oxygen barrier properties to withstand harsher conditions (10^–3^–10^–6^ g m^–2^ day^–1^).^59^ Further improvements in barrier materials can significantly enhance indoor device stability, underscoring the suitability of flexible substrates for reliable indoor operation (see Figure 11E, left). Flexible glass also represents a viable option for conformal, though not fully flexible, applications.

Therefore, stability and lifetime enhancement strategies must be designed with these life-cycle considerations in mind to ensure compatibility with end-of-life and sustainability requirements.

Flexible devices should also undergo standardized bending tests under realistic ambient conditions, with performance measurement over 1000 bending cycles under 1% strain. Both encapsulated and unencapsulated devices should be tested under specified environmental conditions, like illumination (dark/illumination) and biasing (open-circuit/MPPT for dark/illumination).^60^ However, future standardized mechanical stability tests for IPVs should also account for the interconnections between the PV component and other electronic elements, as failure at these interfaces can lead to complete system malfunction. This is especially critical for wearable applications, where devices are continuously subjected to deformation caused by body movements. For wearables, continuous body-induced deformation demands higher bending endurance; Epishine, for instance, tested devices over 10000 bending cycles.

Finally, after production, a device typically undergoes a shelf life period before shipment and use. It then operates under its intended application, environmental, and illumination conditions. At the end of its service life, the product must be recycled or partially reused in compliance with relevant regulations.

With real world performance and lifetime requirements defined, the remaining question is manufacturability and deployment—how these constraints play out in real products and assembly flows, as highlighted by the following industrial insights.

## Industrial Insights—Deployment Challenges and Opportunities for OPV-Based IPVs

Indoor deployments expose PV modules to wide variations in color temperature, incidence angle, and mounting geometry, closely reflecting the realistic hybrid-lighting framework outlined above. OPVs—with their flexibility and spectral tunability—are well suited to such hybrid conditions, motivating light-coupling films and plug-and-play connectors for scalable, automated IoT integration.

While lead-halide perovskites already deliver outstanding indoor PCEs and offer strong prospects for flexibility, spectral tunability, and wide-angle response—with a rapidly growing commercial push for perovskite IPVs— the most deployment-ready body of evidence currently comes from flexible OPV modules, which are already commercialized in indoor IoT products and supported by industrial-scale stability and integration data.

Lighting color temperatures generally vary depending on the installation environment. For example, warm white light (2700–3000 K) is predominantly used in residential spaces across most countries. In contrast, neutral white (3500–4100 K) is commonly used in offices, again in many countries except Japan, where cool white light (5000–6500 K) is more prevalent, even in offices. Cool white lighting is also widely used in industrial environments, due to its high brightness and resemblance to daylight. For the IoT devices powered by PV modules, this light spectrum variation shows that there is no standard spectrum applicable to all IoT device deployment scenarios, particularly when hybrid light is involved (Figure 12).

**Figure 12:**
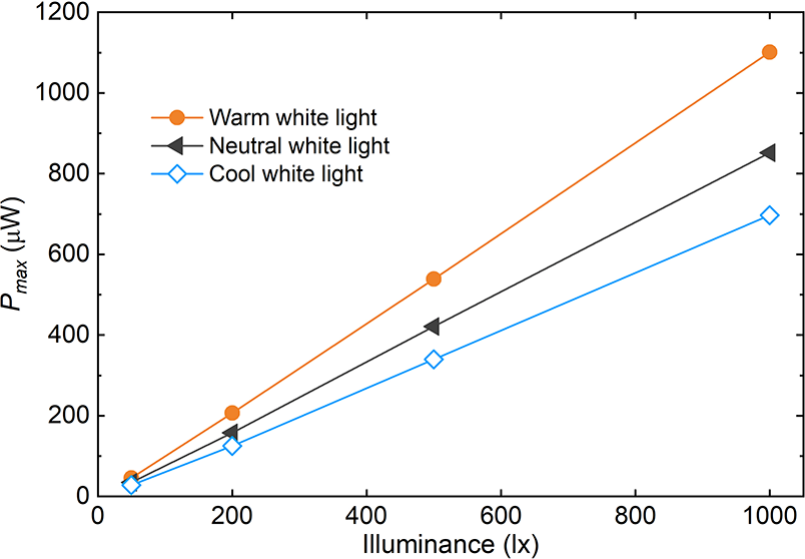
Performance of a DKT OPV module from Dracula technologies under three different color temperature conditions (shown here as an illustrative manufacturer example).

Moreover, the position of the IoT device within a building is also a critical factor, as the PV module performance is directly influenced by the angle of light incidence, regardless of the light spectrum (Figure 13A). For example, a PV module powering an IoT sensor mounted on a vertical surface typically receives illumination at an oblique incidence angle, generally between 40° and 60°, which significantly affects its response. Also, a sensor mounted on the ceiling (Figure 13B), such as those used for smoke detection or environmental monitoring, may be exposed to minimal direct illumination, depending primarily on diffuse and reflected light from surrounding surfaces.

**Figure 13:**
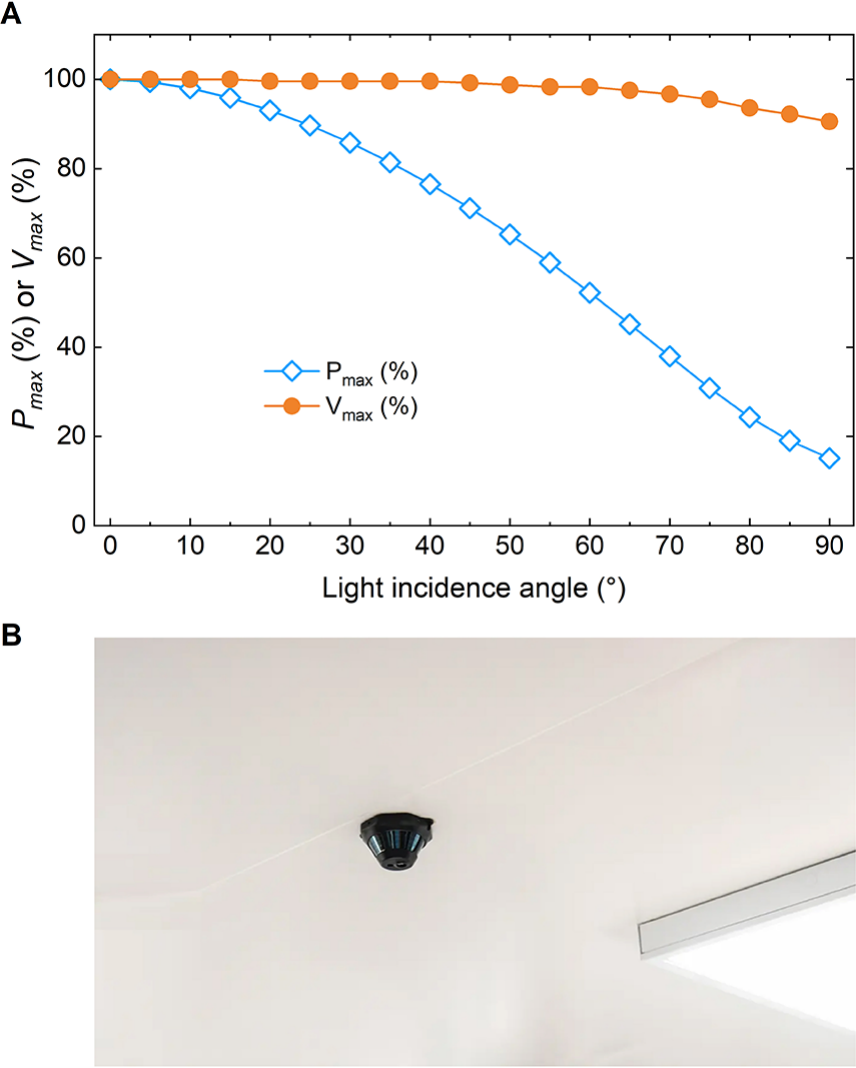
(A) Impact of light incidence angle on the PV module’s performance. (B) Example (Orioma’s LOBX infrared sensor, ceiling-mounted, powered by LAYER OPV technology) illustrating a ceiling-mounted use case.

Finally, in tracking applications powered by PV modules, the tags can be mounted to movable assets such as shipping containers, industrial machinery, or even animals. In such applications, the PV modules may be exposed to indoor environments with artificial or hybrid lighting, as well as outdoor conditions where light intensity can vary significantly. The efficiency of energy harvesting, and therefore the reliability of the tracking system, strongly depends on the intensity and spectral composition of the incident light.

All the points mentioned above highlight the challenges in achieving widespread deployment of self-powered IoT devices. To overcome these challenges in OPV technology, three main improvements are crucial:

1. **Development of a New Generation of OPV Active Materials**: New OPV active materials have been designed to broaden the absorption spectrum and optimize energy harvesting across diverse environments (indoor, hybrid, and outdoor), while maintaining excellent sensitivity under low-light environments. This innovation is particularly important as IoT devices continue to extend their communication range, reaching several kilometers in applications such as LoRaWAN and thus require highly efficient power sources capable of operating reliably under varying illumination levels and spectral composition.
2. **Enhancement of Light-Coupling at the OPV Surface Either by Using a Light-Coupling Substrate or by Printing a Microstructured Film on the Module Surface**: These solutions help reduce reflection and enhance light coupling. Another advantage lies in the use of these films, which enhance the mechanical durability of OPV panels by protecting them from scratches and surface wear. In addition, these films help the devices blend more seamlessly into their environment, improving their visual discretion. As one reported example, manufacturer testing of a microstructured film printed on the OPV surface has shown an average increase of ∼ 8% in light-coupling efficiency under specific illumination and geometry conditions; this value is provided here as an illustrative case rather than a universal performance gain (Figure 14). Here, “optical texturing” refers to a module-level microstructured surface/overlay film that reduces reflection and improves angular light coupling under oblique/diffuse indoor illumination (distinct from texturing of the photoactive layer).
3. **Simplifying Connections for Organic PV Modules**: Electro-mechanical integration is another critical bottleneck for scaling OPV modules with IoT devices. Producing hundreds of thousands of IoT device units requires a high-throughput automated assembly line, yet current processes struggle with handling flexible, ultrathin modules and connecting them to the rest of the electronics. Currently, the available connection solutions (such as soldering, crimping) are insufficient for large-scale IoT production. Developing reliable plug-and-play electrical connections and mechanical attachment methods remains an unresolved challenge.

**Figure 14:**
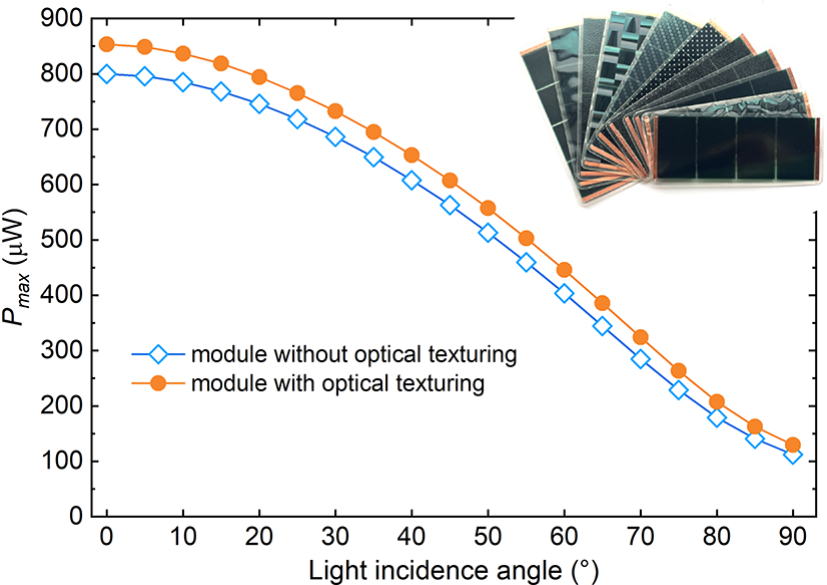
Impact of optical texturing on light coupling and OPV module performance. A microstructured light-coupling film applied on top of the OPV module redistributes incident light, reducing reflection and improving angular acceptance under indoor illumination. The inset shows representative surface microstructures and resulting aesthetic appearances. Reported performance changes correspond to manufacturer testing under specific illumination and geometry conditions.

These industrial insights reinforce the broader message of this Perspective: progress in IPVs depends as much on context-resolved testing and system codesign as on materials innovation. By aligning real-scene characterization, module-to-PMIC integration, and circular-by-design materials, industry and research together can turn hybrid lighting from a variability challenge into a reproducible design space. This convergence between real-world conditions and laboratory precision defines the pathway toward reliable, sustainable, and manufacturable IPV-powered electronics.

## Outlook

Indoor PV will scale fastest when assessed as a deployment-defined system rather than a cell-only PCE exercise. A practical next step for the community is a minimal “starter kit”: (i) a small library of device-plane hybrid reference scenes defined by spectrum, illuminance, geometry/angles, and representative light/dark sequences, including at least one LED-only benchmark anchored at 200 lx for comparability; (ii) explicit reporting of module–PMIC–storage, including the number of series-connected cells, *V*_OC_/*V*_MPP_, PMIC operating window, MPPT approach (and where possible the energy delivered at the PMIC output); and (iii) application-level metrics such as energy-per-uplink, energy-per-inference, and tasks per day, evaluated alongside real-world operational stability. A logical next step toward energy-rating standards is to report these metrics per reference indoor lighting condition, so a defined indoor condition maps to energy delivered at the PMIC output and sustained task throughout under a stated duty cycle and light/dark profile. Over time, this can evolve into a simple, comparable rating that links reference indoor scenes to expected functionality and lifetime for IPV-powered IoT nodes, accelerating deployments in buildings, logistics, and wearables while reducing primary-battery use, e-waste, and enabling circular-by-design, life-cycle-aware comparisons.

## References

[1] Number of connected IoT devices growing 14% to 21.1 billion. https://iot-analytics.com/number-connected-iot-devices/ (accessed 2025–11–02).

[2] Position Paper – Access to facilities and expertise. https://www.enables-project.eu/outputs/position-paper/ (accessed 2025–02–01).

[3] Mathews I., Kantareddy S. N., Buonassisi T., Peters I. M. (2019). Technology and Market
Perspective for Indoor Photovoltaic Cells. Joule.

[4] Wojciechowski K., Forgács D. (2022). Commercial Applications of Indoor Photovoltaics Based
on Flexible Perovskite Solar Cells. ACS Energy
Lett.

[5] Guo Z., Jena A. K., Miyasaka T. (2023). Halide Perovskites for Indoor Photovoltaics:
The Next Possibility. ACS Energy Lett..

[6] Chakraborty A., Lucarelli G., Xu J., Skafi Z., Castro-Hermosa S., Kaveramma A. B., Balakrishna R. G., Brown T. M. (2024). Photovoltaics for
Indoor Energy Harvesting. Nano Energy.

[7] Grandhi G. K., Koutsourakis G., Blakesley J. C., De Rossi F., Brunetti F., Öz S., Sinicropi A., Parisi M. L., Brown T. M., Carnie M. J., Hoye R. L. Z., Vivo P. (2025). Promises and Challenges
of Indoor Photovoltaics. Nat. Rev. Clean Technol..

[8] Hoye R. L. Z., Koutsourakis G., Freitag M., Jehl Li-Kao Z., Österberg T., Aliwell S., Bellanger M., Brown T. M., Brunetti F., Carnie M. J., Chakraborty A., Grancini G., Kärhä P., Kauer M., Kirchartz T., Lin C.-T., Lira-Cantú M., Long Y.-S., Öz S., Raga S. R., Saucedo E., Vivo P., Leow S. W., Wojciechowski K., Zampetti A., Zhou R., Züfle S., Burwell G. (2025). Reaching a Consensus on Indoor Photovoltaics Testing. Joule.

[9] Seunarine K., Haymoor Z., Spence M., Burwell G., Kay A., Meredith P., Armin A., Carnie M. (2024). Light Power Resource
Availability for Energy Harvesting Photovoltaics for Self-Powered
IoT. J. Phys. Energy.

[10] Mainville M., Leclerc M. (2020). Recent Progress on
Indoor Organic Photovoltaics: From
Molecular Design to Production Scale. ACS Energy
Lett..

[11] Politi B., Parola S., Gademer A., Pegart D., Piquemil M., Foucaran A., Camara N. (2021). Practical PV Energy
Harvesting under
Real Indoor Lighting Conditions. Solar Energy.

[12] Jarosz G., Signerski R. (2025). Optimal Bandgap
of a Single-Junction Photovoltaic Cell
for the Mobile Internet-of-Things. iScience.

[13] Proctor C. M., Nguyen T.-Q. (2015). Effect of Leakage Current and Shunt Resistance on the
Light Intensity Dependence of Organic Solar Cells. Appl. Phys. Lett..

[14] Reynaud C. A., Clerc R., Lechêne P. B., Hébert M., Cazier A., Arias A. C. (2019). Evaluation of Indoor
Photovoltaic
Power Production under Directional and Diffuse Lighting Conditions. Solar Energy Materials and Solar Cells.

[15] Lee C.-P., Lin C.-A., Wei T.-C., Tsai M.-L., Meng Y., Li C.-T., Ho K.-C., Wu C.-I., Lau S.-P., He J.-H. (2015). Economical Low-Light
Photovoltaics by Using the Pt-Free Dye-Sensitized
Solar Cell with Graphene Dot/PEDOT:PSS Counter Electrodes. Nano Energy.

[16] Müller M., Wienold J., Walker W. D., Reindl L. M. (2009). Characterization
of Indoor Photovoltaic Devices and Light. 2009
34th IEEE Photovoltaic Specialists Conference (PVSC).

[17] Apostolou G., Reinders A., Verwaal M. (2016). Comparison of the Indoor
Performance
of 12 Commercial PV Products by a Simple Model. Energy Science & Engineering.

[18] Ambient Photonics presents indoor bifacial solar cell tech. pv magazine International. https://www.pv-magazine.com/2024/01/05/ambient-photonics-presents-indoor-bifacial-solar-cell-tech/ (accessed 2025–11–01).

[19] IEC TS 62607-7-2:2023. https://webstore.iec.ch/en/publication/61819 (accessed 2025–02–01).

[20] Guillén C. (2024). Evaluating
Photovoltaic Conversion Performance under Artificial Indoor Lighting. Electronics.

[21] Carvalho C., Paulino N. (2013). Start-up Circuit for Low-Power Indoor
Light Energy
Harvesting Applications. Electron. Lett..

[22] Dallago E., Lazzarini Barnabei A., Liberale A., Torelli G., Venchi G. (2016). A 300-mV Low-Power
Management System for Energy Harvesting Applications. IEEE Transactions on Power Electronics.

[23] AEM10920. E-peas. https://e-peas.com/product/aem10920/ (accessed 2025–11–13).

[24] Azlor M., Martinez B., Gasulla M., Reverter F. (2025). Study of the Applicability
and Limitations of the FOCV Technique for Indoor Low-Area PV Cells. IEEE Transactions on Instrumentation and Measurement.

[25] Kay A. M., Ahmed S. N., Burridge N., Riley D. B., Armin A., Sandberg O. J., Haymoor Z., Carnie M. J., Meredith P., Burwell G. (2025). Optimising Photovoltaic
Modules for Indoor Energy-Harvesting
Systems. J. Phys. Energy.

[26] Dadu M., Kapoor A., Tripathi K. N. (2002). Effect
of Operating Current Dependent
Series Resistance on the Fill Factor of a Solar Cell. Solar Energy Materials and Solar Cells.

[27] Meroni S. M. P., Hooper K. E. A., Dunlop T., Baker J. A., Worsley D., Charbonneau C., Watson T. M. (2020). Scribing Method for Carbon Perovskite
Solar Modules. Energies.

[28] Rakocevic L., Schöpe G., Turan B., Genoe J., Aernouts T., Haas S., Gehlhaar R., Poortmans J. (2020). Perovskite
Modules with 99% Geometrical Fill Factor Using Point Contact Interconnections
Design. Progress in Photovoltaics: Research
and Applications.

[29] Di
Giacomo F., Castriotta L. A., Kosasih F. U., Di Girolamo D., Ducati C., Di Carlo A. (2020). Upscaling Inverted Perovskite Solar
Cells: Optimization of Laser Scribing for Highly Efficient Mini-Modules. Micromachines.

[30] Michaels H., Rinderle M., Benesperi I., Freitag R., Gagliardi A., Freitag M. (2023). Emerging Indoor Photovoltaics for Self-Powered and
Self-Aware IoT towards Sustainable Energy Management. Chem. Sci..

[31] Liu X., Sanchez-Sinencio E. (2015). A Highly Efficient
Ultralow Photovoltaic Power Harvesting
System With MPPT for Internet of Things Smart Nodes. IEEE Trans. Very Large Scale Integr. Syst..

[32] Michaels H., Rinderle M., Freitag R., Benesperi I., Edvinsson T., Socher R., Gagliardi A., Freitag M. (2020). Dye-Sensitized Solar Cells under Ambient Light Powering
Machine Learning: Towards Autonomous Smart Sensors for the Internet
of Things. Chem. Sci..

[33] Flores-Diaz N., Rossi F. D., Keller T., Morritt H., Bassart Z. P., Lopez-Rubio A., Fabra M. J., Freitag R., Gagliardi A., Fasulo F., Muñoz-García A. B., Pavone M., Lomeri H. J., Alonso S. S., Grätzel M., Brunetti F., Freitag M. (2025). Unlocking High-Performance Photocapacitors
for Edge Computing in Low-Light Environments. Energy Environ. Sci..

[34] Kimura N., Jolly V., Latifi S. (2006). Energy Restrained
Data Dissemination
in Wireless Sensor Networks. International Journal
of Distributed Sensor Networks.

[35] Moosmann J., Giordano M., Vogt C., Magno M. (2023). TinyissimoYOLO: A Quantized,
Low-Memory Footprint, TinyML Object Detection Network for Low Power
Microcontrollers. 2023 IEEE 5th International
Conference on Artificial Intelligence Circuits and Systems (AICAS).

[36] Zhang D., Stojanovic M., Ren Y., Cao Y., Eickemeyer F. T., Socie E., Vlachopoulos N., Moser J.-E., Zakeeruddin S. M., Hagfeldt A., Grätzel M. (2021). A Molecular
Photosensitizer Achieves
a Voc of 1.24 V Enabling Highly Efficient and Stable Dye-Sensitized
Solar Cells with Copper­(II/I)-Based Electrolyte. Nat Commun.

[37] Lee H. K. H., Barbé J., Meroni S. M. P., Du T., Lin C.-T., Pockett A., Troughton J., Jain S. M., De Rossi F., Baker J., Carnie M. J., McLachlan M. A., Watson T. M., Durrant J. R., Tsoi W. C. (2019). Outstanding Indoor
Performance of Perovskite Photovoltaic Cells – Effect of Device
Architectures and Interlayers. Solar RRL.

[38] Digitalization and Energy – Analysis. IEA. https://www.iea.org/reports/digitalisation-and-energy (accessed 2025–10–31).

[39] The Global E-waste Monitor 2024. E-Waste Monitor. https://ewastemonitor.info/the-global-e-waste-monitor-2024/ (accessed 2025–10–31).

[40] Paul D., Pechancová V., Saha N., Pavelková D., Saha N., Motiei M., Jamatia T., Chaudhuri M., Ivanichenko A., Venher M., Hrbáčková L., Sáha P. (2024). Life Cycle Assessment of Lithium-Based Batteries: Review
of Sustainability Dimensions. Renewable and
Sustainable Energy Reviews.

[41] Machala M. L., Chen X., Bunke S. P., Forbes G., Yegizbay A., de Chalendar J. A., Azevedo I. L., Benson S., Tarpeh W. A. (2025). Life Cycle
Comparison of Industrial-Scale Lithium-Ion Battery Recycling and Mining
Supply Chains. Nat Commun.

[42] Sasitharan K., Freitag M. (2025). From Zombies to Smart
Devices: The Evolution of Dye-Sensitized
Solar Cells for IoT Applications. ACS Appl.
Energy Mater..

[43] Parisi M. L., Maranghi S., Vesce L., Sinicropi A., Di Carlo A., Basosi R. (2020). Prospective Life Cycle Assessment
of Third-Generation Photovoltaics at the Pre-Industrial Scale: A Long-Term
Scenario Approach. Renewable and Sustainable
Energy Reviews.

[44] Cao Y., Liu Y., Zakeeruddin S. M., Hagfeldt A., Grätzel M. (2018). Direct Contact
of Selective Charge Extraction Layers Enables High-Efficiency Molecular
Photovoltaics. Joule.

[45] Chen R.-T., Liao C.-F. (2014). Evaluation and Optimization
to Recycle Used TiO_2_ Photoelectrode for Dye-Sensitized
Solar Cells. International Journal of Photoenergy.

[46] Shen Z., Eickemeyer F. T., Gao J., Pfeifer L., Bradford D., Freitag M., Zakeeruddin S. M., Grätzel M. (2023). Molecular
Engineering of Low-Cost, Efficient, and Stable Photosensitizers for
Dye-Sensitized Solar Cells. Chem.

[47] Omazic A., Oreski G., Halwachs M., Eder G. C., Hirschl C., Neumaier L., Pinter G., Erceg M. (2019). Relation between Degradation
of Polymeric Components in Crystalline Silicon PV Module and Climatic
Conditions: A Literature Review. Solar Energy
Materials and Solar Cells.

[48] RICOH EH DSSC solid-state dye-sensitized cell product catalog, https://industry.ricoh.com/en/-/Media/Ricoh/Sites/industry/dye-sensitized-solar-cell/pdf/dye-sensitized-solar-cell-en.pdf (accessed 2026–01–06).

[49] Lightricity Ltd. Lightricity Ltd. https://lightricity.co.uk/4evertrack (accessed 2026–01–06).

[50] Lightricity Ltd. Lightricity Ltd. https://lightricity.co.uk/4evairsense (accessed 2026–01–06).

[51] Our LAYER® Technology|Dracula Technologies. https://dracula-technologies.com/technology-layer/ (accessed 2026–01–06).

[52] De
Rossi F., Pontecorvo T., Brown T. M. (2015). Characterization
of Photovoltaic Devices for Indoor Light Harvesting and Customization
of Flexible Dye Solar Cells to Deliver Superior Efficiency under Artificial
Lighting. Applied Energy.

[53] Xu J., Podapangi S. K., Reddy S. H., Castriotta L. A., Di Carlo A., Brown T. M. (2023). Key Parameters
and Thresholds Values
for Obtaining High Performance Perovskite Solar Cells Indoors from
Full Br Compositional and Bandgap Engineering. ACS Appl. Energy Mater..

[54] Müller D., Jiang E., Campos Guzmán L., Rivas Lázaro P., Baretzky C., Bogati S., Zimmermann B., Würfel U. (2024). Ultra-Stable ITO-Free Organic Solar Cells and Modules
Processed from Non-Halogenated Solvents under Indoor Illumination. Small.

[55] Chen C.-H., He X.-Y., Qin R.-H., Wang K.-L., Huang L., Jin R.-J., Chen X., Bian Z.-K., Yang Y.-T., Jin K., Chen J., Xia Y., Yavuz I., Wang Z.-K. (2025). Reliable
Perovskite Indoor Photovoltaics for Self-Powered Devices. Natl Sci Rev.

[56] Epishine’s MultiCell_Data_Sheet_1.6.6. https://www.epishine.com/hubfs/MultiCell_Data_Sheet_1.6.6.pdf (accessed 2025–12–27).

[57] Epishine’s OneCell_Data_Sheet_1.4.4. https://www.epishine.com/hubfs/OneCell_Data_Sheet_1.4.4.pdf (accessed 2025–12–27).

[58] Polyzoidis C., Rogdakis K., Kymakis E. (2021). Indoor Perovskite Photovoltaics for
the Internet of Things—Challenges and Opportunities toward
Market Uptake. Advanced Energy Materials.

[59] Sutherland L. J., Weerasinghe H. C., Simon G. P. (2021). A Review on Emerging Barrier Materials
and Encapsulation Strategies for Flexible Perovskite and Organic Photovoltaics. Advanced Energy Materials.

[60] Fukuda K., Sun L., Du B., Takakuwa M., Wang J., Someya T., Marsal L. F., Zhou Y., Chen Y., Chen H., Silva S. R. P., Baran D., Castriotta L. A., Brown T. M., Yang C., Li W., Ho-Baillie A. W. Y., Österberg T., Padture N. P., Forberich K., Brabec C. J., Almora O. (2024). A Bending
Test Protocol for Characterizing
the Mechanical Performance of Flexible Photovoltaics. Nat Energy.

